# Single-cell combined transcriptome probes prognostic mechanisms of sialylation-related genes in cervical cancer

**DOI:** 10.3389/fonc.2025.1534247

**Published:** 2025-04-25

**Authors:** Gulinigaer Muhetaer, Xinyi He, Chenqing Yang, Fenglan Guo, Ruifang An

**Affiliations:** Department of Obstetrics and Gynecology, The First Affiliated Hospital of Xi’an Jiaotong University, Xi’an, Shaanxi, China

**Keywords:** cervical cancer, sialylation, prognostic features, immunotherapy, mechanisms

## Abstract

**Introduction:**

Sialylation has been linked to cervical dysplasia, while its involvement in cervical cancer is uncertain. Hence , the aim of this study was to develop a prognostic model based on sialylation-related characteristics for cervical cancer patients and investigate how sialylation-related genes are altered in cervical cancer via analyses of transcriptome and single-cell RNA sequencing (scRNA-seq) data.

**Methods:**

The current work incorporated 4 transcriptome datasets relevant to cervical cancer (including scRNA-seq) and 110 sialylation-related genes (SRGs). Initially, differentially expressed SRGs (DE-SRGs) were discovered by differential expression analysis, among other methods. Subsequently, least absolute shrinkage and selection operator (LASSO) and Cox regression analysis was applied using DE-SRGs to detect prognostic genes and build prognostic models. Next, independent prognosis test was conducted, and a nomogram model was built using clinical characteristics and risk scores. Meanwhile, scRNA-seq was applied to examine the cellular composition and cell-to-cell regulation in cervical cancer vs normal group, and key cells were determined via prognostic genes and their differentiation process was investigated. Finally, the immunological microenvironment, mutant genes, and medication sensitivity were assessed. Clinical samples were taken to assess the expression of prognostic genes by quantitative reverse transcriptase PCR (qRT-PCR).

**Results:**

First, we detected 19 DE-SRGs related with sialylation. Three prognostic genes, GALNT12, GCNT4, and NPL, were discovered by LASSO cox regression. A risk model constructed with prognostic genes revealed that patients in high-risk group had a much poorer survival rate than those in group with low risk. Meanwhile, low-risk cervical cancer patients were more likely to respond to immunotherapy and chemotherapy, depending on immunology, tumor microenvironment, and drug sensitivity. ScRNA-seq data suggests that the expression of prognostic genes was higher in key cells, macrophages and fibroblasts, and played a more critical role in cervical cancer. The findings from qRT-PCR demonstrated that GCNT4 and NPL were considerably overexpressed in the cervical cancer group.

**Discussion:**

In this research, GALNT12, GCNT4 and NPL were discovered as sialylation-related prognostic genes in cervical cancer, providing novel pathways for detection and treatment.

## 1 Introduction

Cervical cancer is one of the most common malignant gynecological tumors, with squamous cell carcinoma being the primary histological type, followed by adenocarcinoma (1). Cervical cancer ranks first among female cancers in 23 countries globally and is the most common cause of death from cancer in women in 36 countries (2). It ranks fourth in terms of incidence and mortality rates among all female cancers worldwide, it accounts for 6.5% of all cancer cases in women and 7.7% of all cancer deaths in women (2). In recent years, although the incidence of cervical cancer has decreased due to the widespread use of the human papillomavirus (HPV) vaccine, the prognosis for patients with advanced cervical cancer remains poor. The most common treatments for cervical cancer are surgery, radiation and chemotherapy (3). In recent years, the application of the tumor microenvironment in targeted therapy has diversified cancer treatment. In cancers such as lung cancer, immune cell profiles aid in early detection and prevention (4), while targeting T-cell exhaustion has become a key research focus (5). For women with metastatic or recurrent cervical cancer, the overall prognosis continues to be poor, with a 5-year survival rate of about 10 to 20% (6). Currently, conventional prognostic indicators for cervical cancer include age, stage, histological type, and lymph node metastasis. While these factors can reflect the patient’s prognostic risk to some extent, their predictive power is limited. Therefore, there is an urgent requirement for the identification of effective prognostic genes to further investigate the pathogenesis of cervical cancer, promote the diagnosis and treatment of cancer of the cervix, and guide the prediction of cervical cancer prognosis.

Sialic acid (N-acetylneuraminic acid) is a carbohydrate found on the surface of mammalian cells and belongs to a family of more than 50 carbohydrates (7). Sialic acid is biosynthesized from N-acetyl-mannosamine and transferred to the termini of glycolipids and glycoproteins by a set of 20 different sialyltransferases (STs) (7). Malignancy is often associated with changes in cellular sialoglycan expression, and could therefore be used as a diagnostic and prognostic biomarker (8). Increased levels of total-sialic acid in the serum have been found in many patients with cancer. Hypersialylation helps tumor cells to grow and metastasize, leading to a poorer prognosis (9). Sialoglycans are recognized by sialic acid-binding receptors on immune cells, including sialic acid-binding immuno globulin like lectins (Siglecs). The sialoglycan-Siglec interaction leads to suppressed immune response because sialic acids are considered self-associated molecular patterns (10). Studies have shown that sialylation plays a crucial role in gynecological cancers such as ovarian, cervical, and endometrial cancers (10). It is responsible for alterations in immune surveillance, apoptosis, cell death, changes in cancer cell surface, and the development, growth and metastasis of the tumor and its microenvironment (11). Many studies have investigated the role of sialic acid in gynecological cancers, with most focusing on ovarian cancer and to a lesser extent endometrial and cervical cancer (12). However, the mechanism of sialylation in cervical cancer prognosis remains unclear, and the role of sialylation will be the subject of further research in cervical cancer prognosis.

Therefore, to comprehensively understand the prognostic value and molecular mechanisms of sialylation-related genes (SRGs) in cervical cancer, this study first utilized the transcriptome data of 174 cervical cancer patients and single-cell sequencing datasets from the TCGA database. Using a range of bioinformatics methods, including differential gene expression analysis and LASSO Cox regression analysis, prognostic genes were identified and a prognostic model was constructed. Subsequently, through further bioinformatics analysis, the expression, function, immune infiltration, and drug sensitivity of sialylation in cervical cancer were comprehensively explored. Meanwhile, by the analysis of single-cell RNA sequencing (scRNA-seq) data, key cell types in cervical cancer were identified, and their differentiation states and cell-cell interactions were investigated to provide important references for revealing their potential roles in cervical cancer.

## 2 Materials and methods

### 2.1 Data source

The Cancer Genome Atlas (TCGA)-Cervical Squamous Cell Carcinoma and Endocervical Adenocarcinoma (CESC), GSE63514 (GPL570), GSE44001 (GPL14951), and scRNA-seq dataset GSE168652 were the 4 transcriptome datasets connected to cervical cancer. Among these, GSE63514, GSE168652 (GPL24676), and GSE44001 from the Gene Expression Omnibus (GEO) database (http://www.ncbi.nlm.nih.gov/geo/) contained 28 cervical cancer and 24 normal samples, 1 cervical cancer and 1 normal sample, and 300 cervical cancer samples with complete survival time and status, respectively. Then, the TCGA-CESC collected from the website of UCSC Xena (http://xena.ucsc.edu/) comprised 174 cervical cancer samples with completed survival information, according to which all samples were randomly divided into a training set (121) and validation set (53) in a 7 to 3 ratio via caret (v 6.0-93) (13) for subsequent analysis. All datasets employed cervical epithelial tissue as their sample type. Ultimately, 110 SRGs (including salivary acid transferase, transporter proteins, and neuraminidase) were extracted from the Molecular Signatures Database (MSigDB, https://www.gsea-msigdb.org/gsea/msigdb) (**Supplementary Table 1**) (14, 15).

### 2.2 Differential expression analysis and functional enrichment

The GSE63514 dataset was analyzed by limma (v 3.54.0) (16) to locate differentially expressed genes (DEGs) (*adj. P* < 0.05 & |log_2_Fold Change (FC)| > 0.5). To visualize DEGs, we used ggplot2 (v 3.4.1) (17) to create a volcano plot and label the top 10 most significant upregulated and downregulated genes. We also used ComplexHeatmap (v 2.15.1) (18) to generate a heatmap displaying the expression patterns of the top 10 upregulated and top 10 downregulated genes, ranked by log_2_FC, across different samples. Besides, DEGs were intersected with SRGs to generate differentially expressed SRGs (DE-SRGs). The clusterProfiler (v 4.2.2) (19) was implemented to assess Gene Ontology (GO) and Kyoto Encyclopedia of Genes and Genomes (KEGG) enrichment analysis for biological processes in DE-SRGs (*adj. P* < 0.05), the top 20 remarkably enriched pathways were visualized. Meanwhile, a protein-protein interaction (PPI) network about DE-SRG proteins was built with the STRING database (https://string-db.org/) (medium confidence >0.4; isolated targets were deleted).

### 2.3 Establishment of prognostic characteristics

To locate the prognostic genes, a univariate Cox analysis for DE-SRGs using survival (v 3.3-1) (20) was first conducted. Assume that the ratio of risk functions at different gene levels does not change over time, and the survival time and survival status of each sample are independent of each other; take the survival time and survival status as dependent variables, and the expression value of each gene as an independent variable; calculate the hazard ratio (HR) of each gene, its 95% confidence interval and *P*-value (HR≠1 & *P* < 0.05). Then, the least absolute shrinkage and selection operator (LASSO) regression analysis was carried out through glmnet (v 4.1-2) (21). Hypothesis one was that there was a linear relationship between gene expression values and survival outcomes; hypothesis two was that the hazard ratio remained constant throughout the follow-up period; hypothesis three was that the survival time and survival status of each sample were independent of each other. The cv.glmnet function was used for 10-fold cross-validation to determine the minimum lambda value. Finally, the genes whose regression coefficients were not penalized to 0 were determined as the prognostic genes related to the survival outcome. Next, risk scores were generated by applying the expression of prognostic genes in training set, validation set, and GSE44001 dataset, as well as risk coefficients in LASSO, using the following formulas:


$$risk\;score=\sum_{i=1}^{n}coef(gene_{i})\times \mathrm{expr}(gene_{i})$$

Where was the risk coefficient, and was the prognostic gene expression. In addition, the cervical cancer samples in three datasets were split into high and low risk groups according to median risk scores. Survival analyses were conducted using the survminer (v 0.4.9) (*P* < 0.05) (22) with a significance level of P < 0.05. The survfit function was used to calculate the differences in survival curves, and the ggsurvplot function was employed to plot the survival probability curves. To assess the survival differences between the high and low-risk groups, the log-rank test was used to evaluate the significance of the differences. Meanwhile, the receiver operating characteristic (ROC) curves at 1, 3, and 5 years were plotted with timeROC (v 0.4) (23) to evaluate the risk model’s prediction ability (Area Under the ROC curve (AUC) > 0.6). Furthermore, different risk curves were drawn to investigate the link between survival status, risk scores, and prognostic gene expression in cervical cancer patients of three datasets.

### 2.4 Independent prognostic analysis

To explore the association between distinct clinical features and prognostic characteristics, survival analyses between two risk groups were conducted with the training set’s various clinical characteristics of cervical cancer patients. In the meantime, the univariate Cox analysis involved risk scores, age, pathologic_Stage, pathologic_T, pathologic_N, histologic_Grade, and HPV type (24) was conducted using survival (v 3.3-1) (20) (*P* < 0.2). After acquiring independent prognostic indicators by multivariate Cox (*P* < 0.2) and proportional hazards (PH) assumption testing (*P* > 0.05), nomogram was created using rms (v 6.5-0) (25) to forecast the 1-, 3-, and 5-year survival of patients with cervical cancer. And calibration curve was subsequently utilized to evaluate the nomogram’s prediction ability.

### 2.5 Functional enrichment and mutation analysis

To further understand the functional pathways involved in prognostic genes, single-gene Gene set enrichment analysis (GSEA) was utilized. In brief, the Spearman correlation of prognostic genes with the remaining portion of the genes was computed separately in training set and ordered in descending order of coefficient size. Then, GSEA was conducted on the sorted genes via clusterProfiler (v 4.2.2) (26), with the KEGG gene set in msigdbr (v 7.5.1) (15) as background gene set (*adj. P* < 0.05). Additionally, the frequency of mutated genes between two risk groups in training set was then calculated with TCGAmutations (v 0.3.0) (27).

### 2.6 Construction of mRNA-miRNA-lncRNA network

The TarBase (http://www.microrna.gr/tarbase) and miRTarBase databases (http://miRTarBase.mbc.nctu.edu.tw/) were utilized to predict miRNAs targeting prognostic genes relying on the NetworkAnalyst platform (https://www.networkanalyst.ca/). A cross-section of prognostic gene-miRNA pairs predicted by the two databases was taken. And the miRNAs in the intersected pairings were extracted to forecast the corresponding lncRNAs using starbase database (https://rnasysu.comencori/). Ultimately, the mRNA-miRNA-lncRNA regulation network was built.

### 2.7 Immunological correlation analysis

To investigate the involvement of immune cells in cervical cancer, the single sample GSEA (ssGSEA) algorithm in GSVA package (v 1.42.0) (28) was implemented to evaluate the functional status of different immune cells in the samples. Gene expression data (FPKM) was ranked, and analysis was performed using kcdf=“Gaussian” to calculate the enrichment scores for each sample in specific gene sets, such as immune cell marker genes. A heatmap was generated to display the differences in immune cell scores between the high-risk and low-risk groups. Next, the Wilcoxon signed rank test was used to compare the differential expression of 28 immune cells between the two risk groups (*adj. P* < 0.05). Meanwhile, the Spearman correlation between prognostic genes and distinct immune cells was assessed with psych (v 2.2.9) (29). Besides, to further comprehend the composition of tumor microenvironment, the stromal score, immune score, and ESTIMATE score of cervical cancer patients in two risk groups were evaluated in training set via estimate (v 1.0.13) (30), and their correlations with risk score was computed. In addition, 48 immune checkpoint molecules retrieved from the literature were employed to examine expression differences between two risk groups in training set (*adj. P* < 0.05) (31). Additionally, to assess the potential response of cervical cancer patients to immunotherapy, dysfunction and exclusion scores of cervical cancer samples in training set were taken from the Tumor Immune Dysfunction and Exclusion (TIDE) website (http://tide.dfci.harvard.edu/), and their correlation with risk score was calculated. At last, to probe differences in chemotherapeutic agent sensitivity among cervical cancer patients in two risk groups, drug semi-inhibitory concentrations (IC50) of 138 chemotherapeutic agents were estimated using pRRophetic (v 0.5) (32) and compared across groups by Wilcoxon signed rank test (*adj. P* < 0.05).

### 2.8 Pre-processing of scRNA-seq data

To generate high-quality data, Seurat (v 5.0.1) (33) initially served to remove cells with fewer than 200 genes and fewer than three cells covered with genes. To determine the final cells and genes chosen for the study, the quality control criteria were set to nFeature_RNA number larger than 300 but less than 5000, nCount_RNA number less than 20,000, and percent.mt less than 20%. The data were then gradually normalized, and highly variable genes were selected using Seurat’s (v 5.0.1) (33) NormalizeData and FindVariableFeatures functions. Next, the data were normalized with the ScaleData function and gravel displayed with the ElbowPlot function to look for principal components (PCs). The Uniform Manifold Approximation and Projection (UMAP) clustering was conducted by applying the screened PCs, with the resolution set at 0.5. Furthermore, the cell population composition was obtained through annotating the clusters with marker genes (34), and bar graphs were generated to illustrate the cell ratio between cervical cancer and normal groups.

### 2.9 Cellular communication and pseudotime analysis

To further comprehend cell relationships, cell communication analysis was conducted independently in cervical cancer and normal groups employing celltalker (v 0.0.7.9000) (35). Subsequently, the expression of prognostic genes was evaluated in all cells, and cells with high expression of prognostic genes were chosen as key cells and submitted to pseudotime analysis via monocle (v 2.26.0) (36) to determine their differentiation state. Meanwhile, the expression of prognostic genes in various differentiation phases of key cells, as well as GSE63514, was observed.

### 2.10 The quantitative reverse transcriptase PCR

Five cancer samples from cervical cancer patients and five samples of normal tissue were gathered from the First Affiliated Hospital of Xi’an Jiaotong University Hospital to confirm the expression of prognostic genes. In order to extract RNA, 50 mg of tissue from each sample was first homogenized using 1 ml of TRIzol reagent (Ambion, USA). After measuring the amount of RNA, reverse transcription was started right away. In short, a reaction system was set up in accordance with the manufacturer’s instructions for the SweScript First Strand cDNA Synthesis Kit (servicebio, China) to generate cDNA. Next, using a CFX96 real-time fluorescence quantitative PCR device, 40 cycles of qPCR amplification were carried out. The primer sequences were displayed in **Table 1**, and the 2^-ΔΔCt^ method was employed to evaluate the prognostic genes’ expression.

**Table 1:** Table of RT-qPCR primer sequence.

Primer	Sequence
GALNT12 F	CCAACAAGAGAGAGGGCCTG
GALNT12 R	CGGAGTTCCCCAGGTATTCG
GCNT4 F	GGGATCCGAGCCGAGAAAC
GCNT4 R	GGAGGTGCTACAGATGGCTG
NPL F	CGGAGGCCTGGAGAAATCAA
NPL R	ATCCTCATGCAGCCACTCAC
GAPDH F	CGAAGGTGGAGTCAACGGATTT
GAPDH R	ATGGGTGGAATCATATTGGAAC

### 2.11 Statistical analysis

The R programming language (v 4.2) was implemented for bioinformatics analyses. Differential expression analysis was conducted using the limma package, assuming that the residuals followed a normal distribution and applied a linear model to fit the data. The Wilcoxon signed rank test was used to compare the data between different groups, and multiple hypothesis correction was performed using the Benjamini-Hochberg method (BH) to control the false discovery rate (FDR) (P < 0.05). The Log-rank test was employed to assess the significance of differences in survival probability curves between the high-risk and low-risk groups.

## 3 Results

### 3.1 The 19 DE-SRGs had a substantial association with sialylation

**Figure 1:**
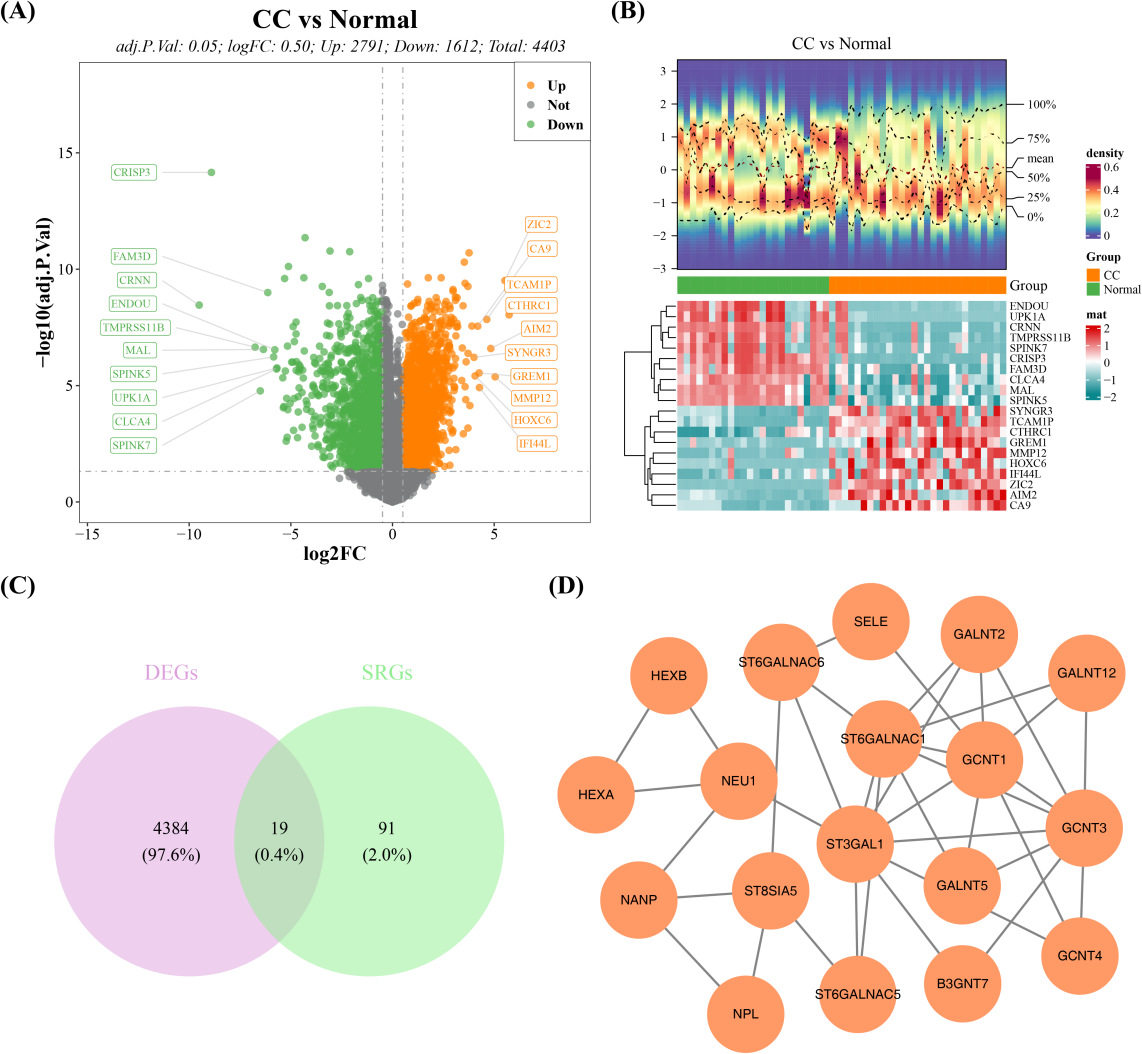
DE-SRGs gene screening and functional analysis in the GSE63514 dataset of cervical cancer. **(A)** Volcano plot of differentially expressed genes between cervical cancer and normal tissue. **(B)** Heatmap of differentially expressed genes between cervical cancer and normal tissue. **(C)** Candidate gene intersection Venn diagram. **(D)** PPI network of candidate genes.

After differential expression analysis in GSE63514 dataset, a total of 4,403 DEGs were detected between cervical cancer and normal groups, of which 2,791 were elevated and 1,612 reduced (**Figures 1A, B**, **Supplementary Table 2**). After taking the intersection of 110 SRGs and 4,403 DEGs, 19 DE-SRGs were acquired (**Figure 1C**). Consistent with our hypothesis, functional enrichment subsequently confirmed that they were primarily linked to 112 KEGG signaling pathways (e.g. Mucin type O-glycan biosynthesis and Glycosphingolipid biosynthesis-ganglio series) and 112 GO items (e.g. sialylation, Golgi stack and glycosyltransferase activity) (**Supplementary Figure 1**, **Supplementary Tables 3, 4**). In the meanwhile, complicated interactions between DE-SRG-encoded proteins, including GALNT12-ST6GALNAC1, GCNT3-ST3GAL1, GCNT4-GCNT3, and NPL-NANP, were discovered through the PPI network (**Figure 1D**).

### 3.2 A risk model constructed around GALNT12, GCNT4, and NPL correctly predicted the survival r ate of cancer patients

**Figure 2:**
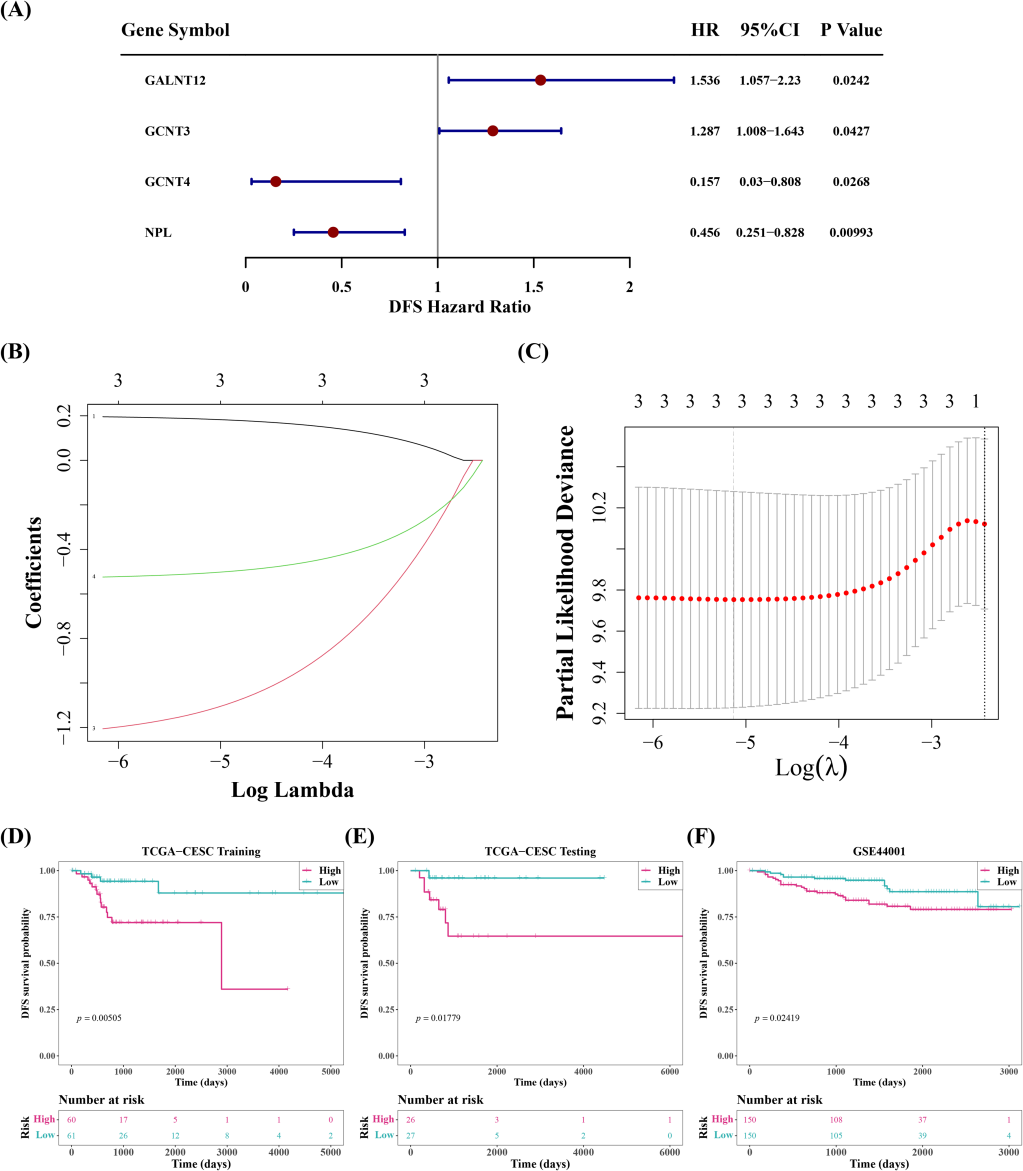
Screen for prognostic genes, classify cervical cancer samples into high-risk and low-risk groups based on these prognostic genes, and perform survival analysis between the groups. **(A)** Forest plot of univariate cox analysis. **(B, C)** Ten-fold cross-validation for adjusting parameters in Lasso analysis and coefficient path plot. **(D)** Kaplan-Meier survival analysis and the number of patients in different groups of the training set. **(E)** Kaplan-Meier survival analysis and the number of patients in different groups of the internal test set. **(F)** Kaplan-Meier survival analysis and the number of patients in different groups of the validation set.

A univariate Cox regression study of 19 DE-SRGs detected 4 prognosis-related genes. GALNT12 and GCNT3 had HRs greater than 1, indicating that they were risk factors for cervical cancer, whereas GCNT4 and NPL had the reverse effect (**Figure 2A**). Further LASSO analysis demonstrated that the lowest model error rate occurred when the minimal lambda was equal to 0.00591. The genes corresponding to this point, GALNT12, GCNT4 and NPL, were defined as prognostic genes (**Figures 2B, C**).

**Figure 3:**
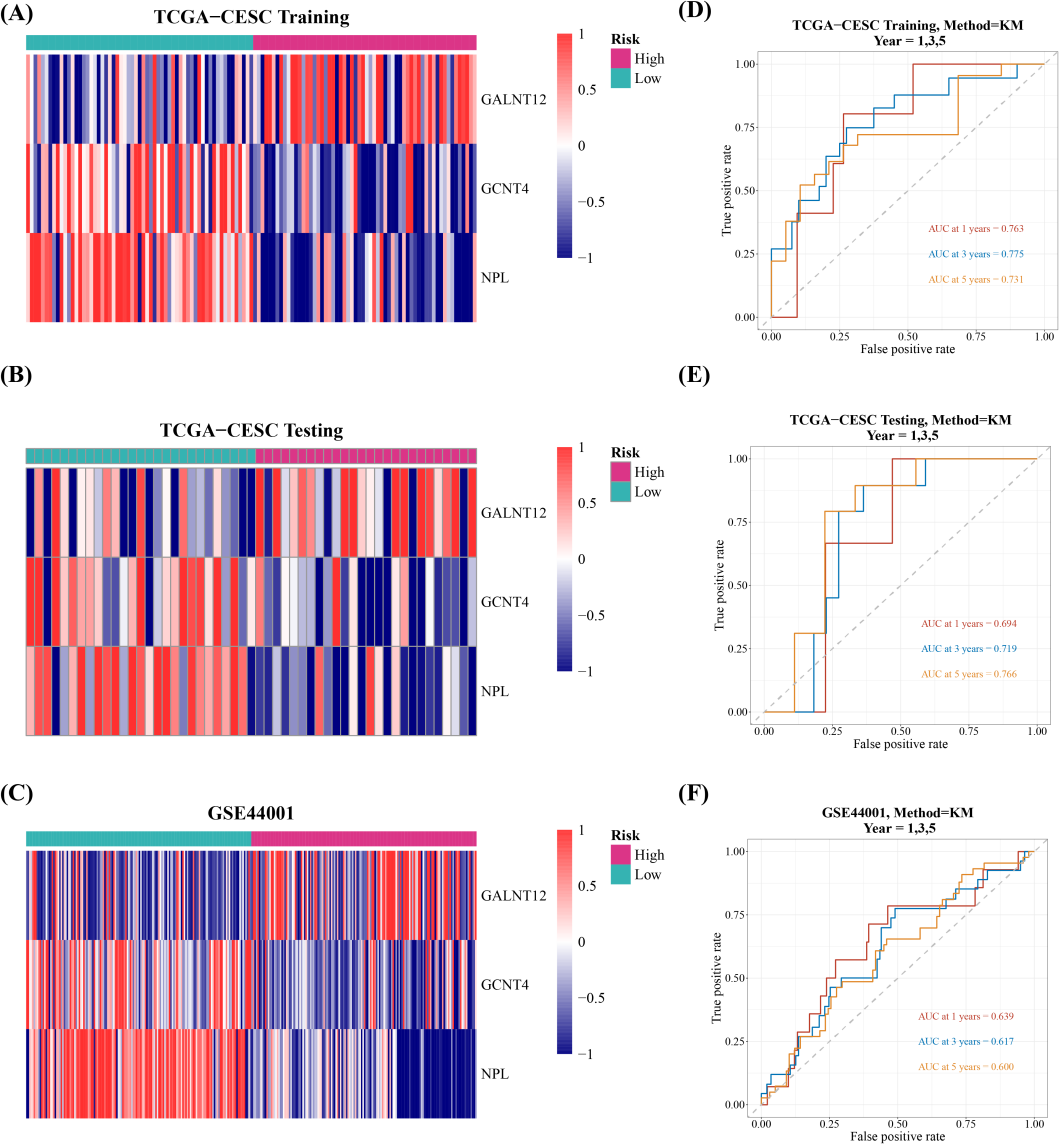
Evaluation and validation of the risk model in the training, test, and validation sets. **(A-C)** Expression levels of prognostic genes in tumor samples. **(D-F)** ROC curves for 1, 3, and 5 years.

Afterwards, the cervical cancer samples were then classified into high and low risk groups, with median risk scores of -1.493621, -1.173702, and -10.86075 in training set, validation set, and GSE44001 dataset, respectively. Between-group survival analysis revealed that patients in high-risk group in all three datasets had considerably worse survival rates than those in low-risk group (**Figures 2D–F**). The risk curves additionally indicated that the number of cervical cancer deaths increased as risk scores rose (**Supplementary Figure 2**). And GALNT12 expression was much higher in high-risk group than GCNT4 and NPL, consistent with the univariate cox results (**Figures 3A–C**). The ROC curves demonstrated that the AUC values of cervical cancer patients were greater than 0.6 in all three datasets for years 1, 3, and 5, implying that prognostic genes established by risk model could be employed to accurately assess the risk of cervical cancer patients (**Figures 3D–F**).

### 3.3 Risk score and HPV typing recognized as independent prognostic factors

**Figure 4:**
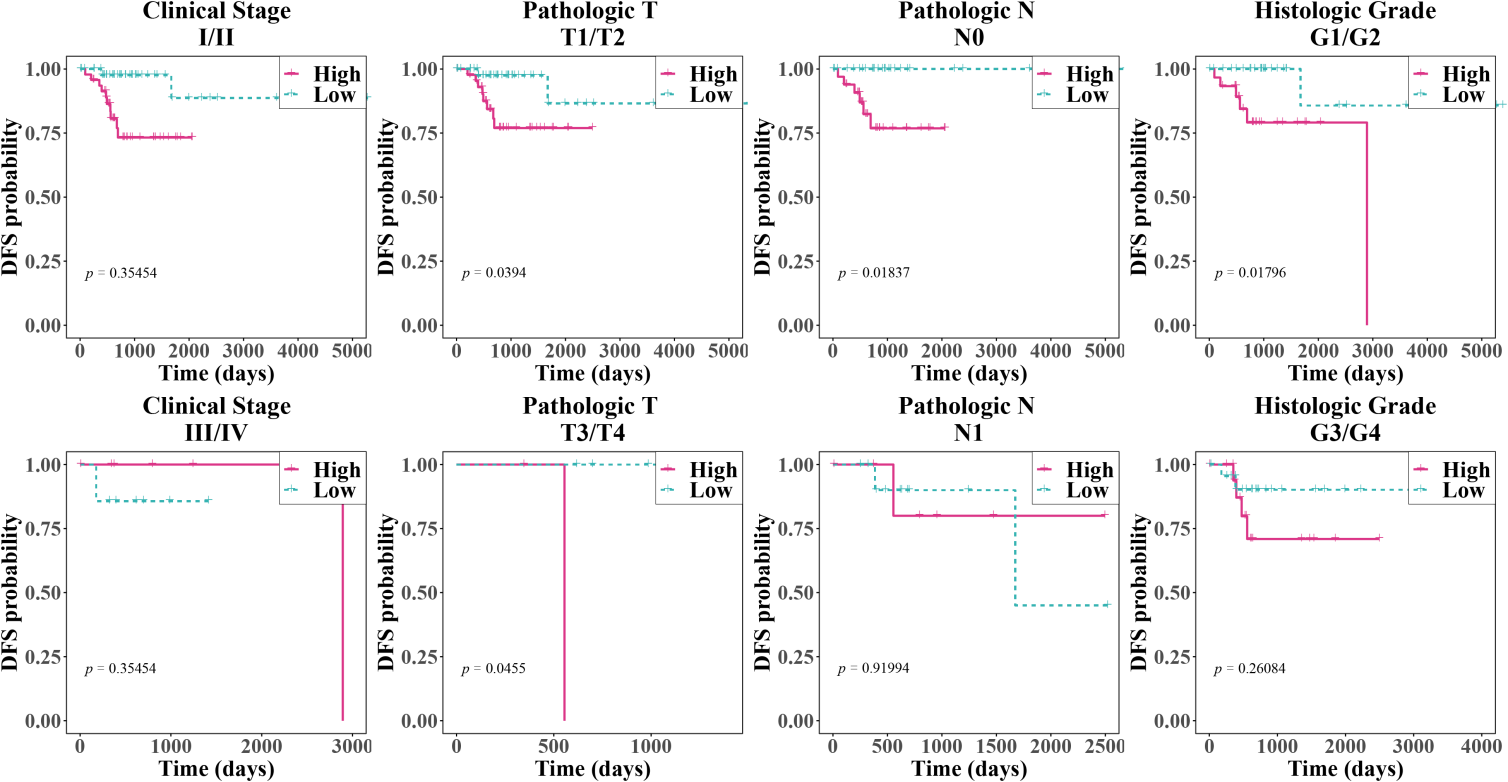
Kaplan-Meier survival curves for patients with different clinical characteristics. The x-axis represented time (in days), and the y-axis represented the disease-free survival probability, with values ranging from 0 to 1. From left to right, the clinical stages, pathological T, pathological N, and histological grades were shown. The pink solid line represented the high-risk group, while the blue dashed line represented the low-risk group. This was used to compare the disease-free survival of patients under different clinical characteristic groupings. A p-value less than 0.05 typically indicated a significant survival difference between the two groups.

**Figure 5:**
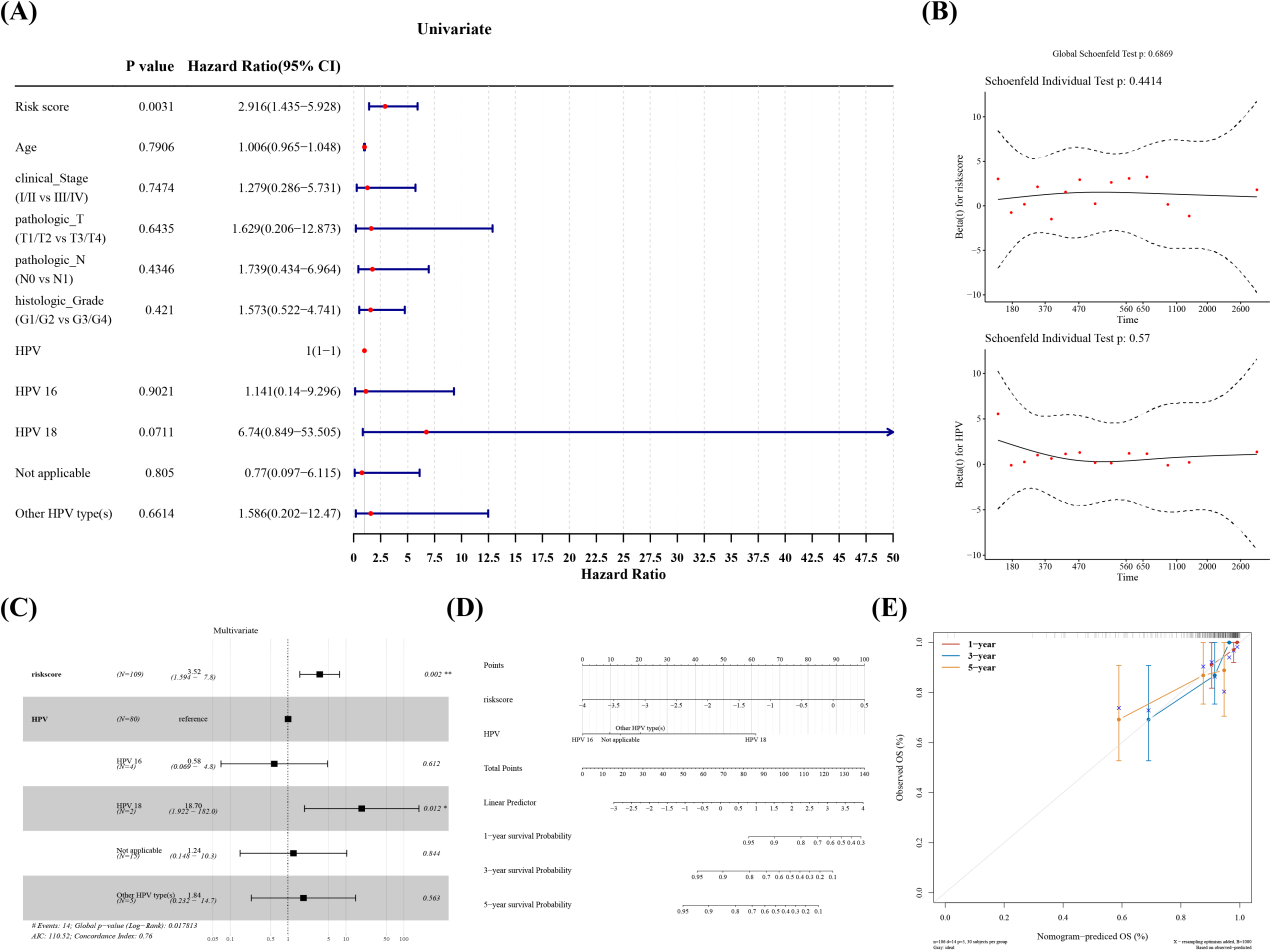
Independent prognostic analysis and construction of the nomogram. **(A)** Forest plot of one-way cox regression clinical indicators. **(B)** GALNT12, GCNT4, NPL Multifactor cox regression PH Hypothesis test. **(C)** Multifactor cox regression forest plot. **(D)** Create a nomogram for predicting the 1-year, 3-year, and 5-year survival rates of cervical cancer patients based on a multivariate Cox regression model. **(E)** Plot a calibration curve to evaluate the performance of the prognostic prediction model.

In several subgroups with diverse clinical features, we noticed substantial survival differences between two risk groups in patients with Pathologic T (T1/T2), Pathologic T (T3/T4), Pathologic N (N0), and Histologic Grade (**Figure 4**, **Table 1**). Furthermore, all clinical variables and risk score, were evaluated in cox regression analysis and PH assumption test, which confirmed that risk score and HPV typing were independent prognostic indicators (**Figures 5A–C**). A nomogram developed on this basis demonstrated that the higher the overall score, the worse the survival rate of cervical cancer patients, and the slope of calibration curve tended to be 1, validating the nomogram’s prediction accuracy (**Figures 5D, E**).

### 3.4 Prognostic genes were involved in multiple functions and molecular regulatory mechanisms

**Figure 6:**
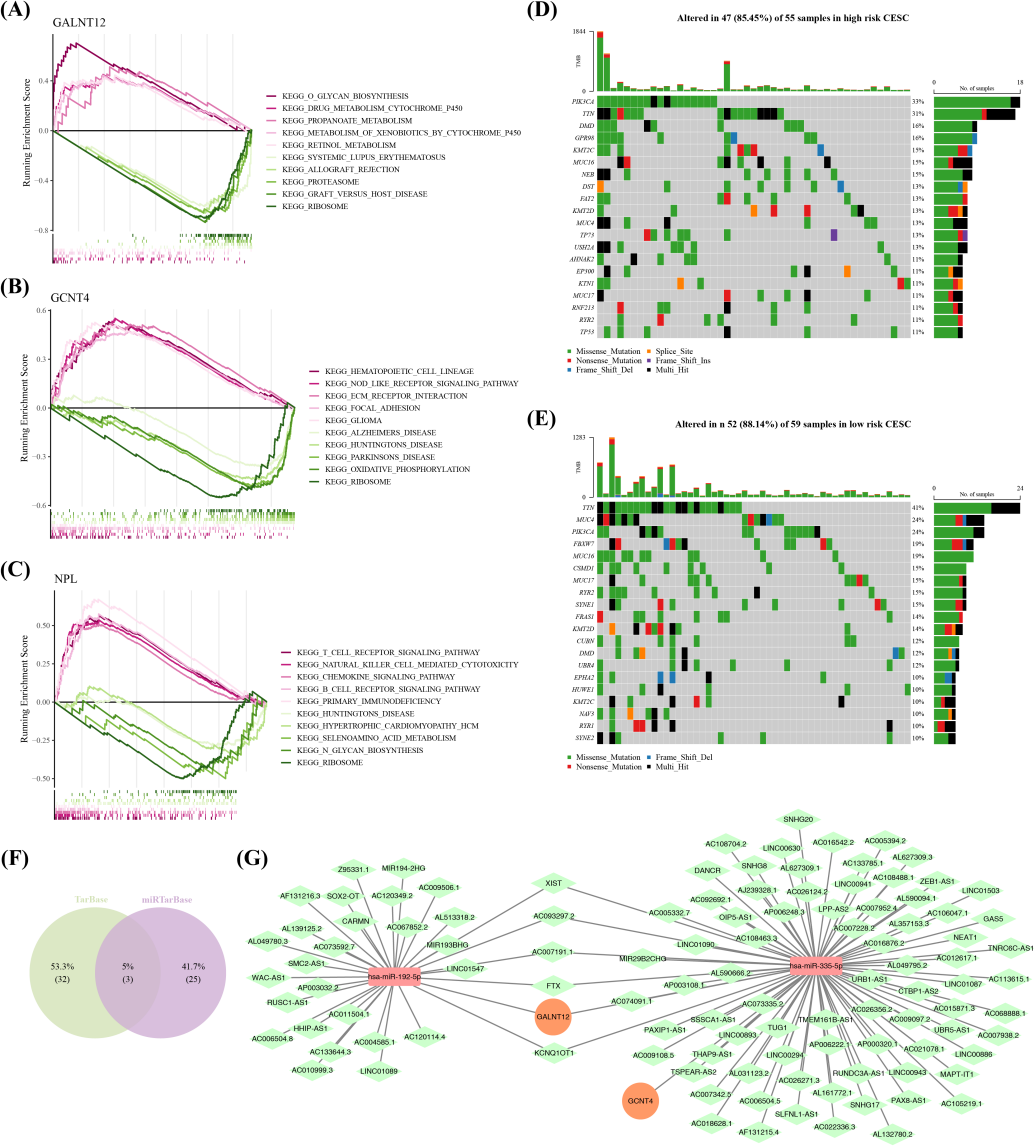
Perform GSEA (Gene Set Enrichment Analysis) to explore the various functions and molecular regulatory mechanisms involved with the prognostic genes. **(A-C)** GSEA enrichment analysis of GALNT12, GCNT4 and NPL. **(D)** Plot a waterfall chart for the top 20 genes with the highest mutation frequencies in the high-risk group. **(E)** Plot a waterfall chart for the top 20 genes with the highest mutation frequencies in the low-risk group. **(F)** Venn plots of miRNA-mRNA relationships obtained from both databases. **(G)** A ceRNA (competitive endogenous RNA) regulatory network,Orange circles represent prognostic genes, red rectangles represent miRNAs, and green rectangles represent lncRNAs.

GSEA outcomes indicated that GALNT12 was primarily associated with glycan biosynthesis and propanoate metabolism, among other things (**Figures 6A**); GCNT4 was primarily associated with the functions of focal adhesion and Alzheimer’s disease (AD) (**Figures 6B**); and NPL was linked to with the pathway regulation of T cell receptor, chemokine signaling pathway, and other pathways (**Figures 6C**). Moreover, mutation analysis discovered that PIK3CAhad the highest mutation rates in high-risk groups, at 33% (**Figure 6D**), which may suggest that mutations in this gene are associated with the invasiveness and poor prognosis of cervical cancer. In contrast, the mutation rate of TTN was the highest in the low-risk group, at and 41% (**Figure 6E**), which may be related to the lower invasiveness and better prognosis of the tumor. After predicting the TarBase and miRTarBase databases, we located two target miRNAs (hsa-mir-192-5p and hsa-mir-335-5p) (**Figure 6F**). The mRNA-miRNA-lncRNA network proved that prognostic genes were involved in the regulation of several non-coding RNAs (no prediction findings for NPL), including GALNT12-hsa-miR-192-5p-LINC01547 and GCNT4-hsa-miR-335-5p-GAS5, among others (**Figure 6G**).

### 3.5 Patients in low risk group might respond more effectively to immunotherapy

**Figure 7:**
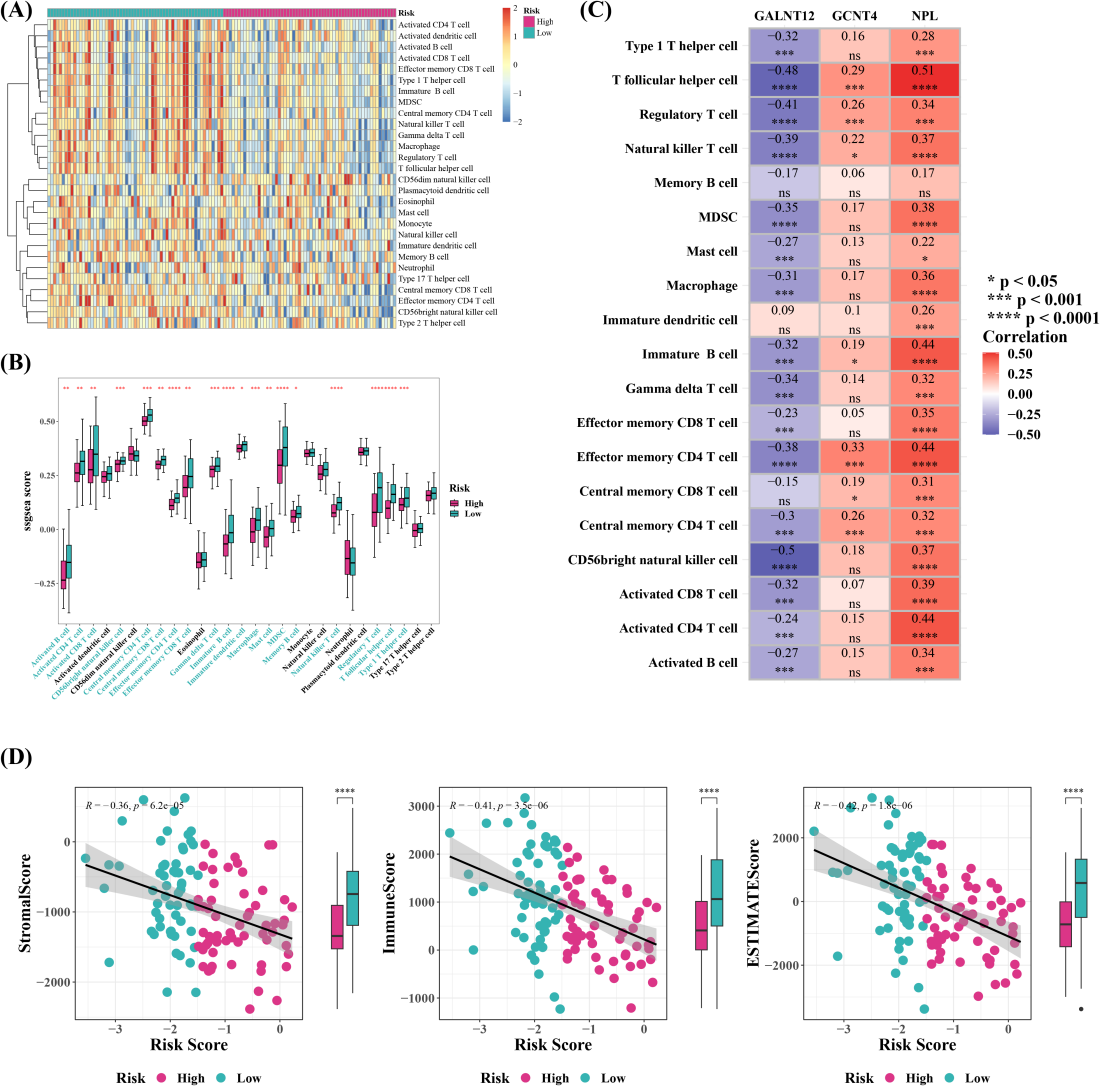
Immune infiltration analysis and ESTIMATE immune characteristic analysis. **(A)** Heatmap of immune infiltration cell enrichment scores based on highand low-risk groups. **(B)** Boxplot of the enrichment scores of 28 types of immune infiltration cells between high-risk and low-risk sample groups. *P < 0.05, **P < 0.01, ***P < 0.001, ****P < 0.0001. The blue labels on the **(C)** Heatmap showing the correlation between differential immune infiltration cells and model genes. ns represented no significance, *P < 0.05, ***P < 0.001, ****P < 0.0001. **(D)** Estimate the StromalScore, ImmuneScore, and ESTIMATEScore for tumor samples based on expression data, and plot the corresponding scatter plots to visualize the relationships.

**Figure 8:**
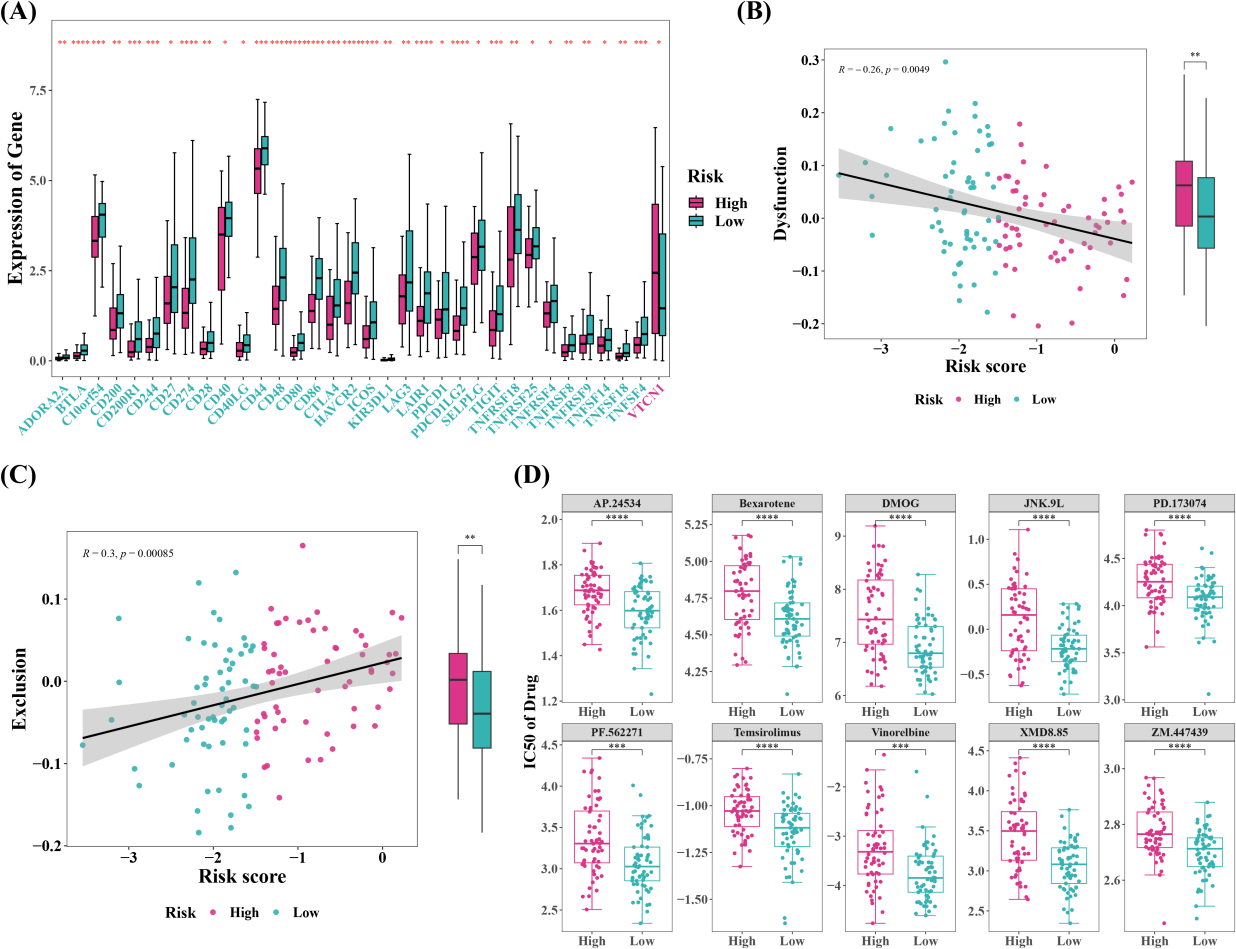
Immune therapy response prediction, and chemotherapy drug sensitivity prediction between high- and low-risk groups. **(A)** Create a boxplot of immune checkpoint molecule expression levels between the high-risk and low-risk groups. *P < 0.05, **P < 0.01, ***P < 0.001, ****P < 0.0001. **(B, C)** Correlation analysis between TIDE (Tumor Immune Dysfunction and Exclusion) scores and risk scores. **P < 0.01. **(D)** top10 Chemotherapy drug IC50 based on boxplots between high and low risk groups. ***P < 0.001, ****P < 0.0001.

The outcomes of immune infiltration demonstrated substantial differences between two risk groups for 19 types of immune cells, including immature dendritic cells and memory B cells, both of which were observed in high levels in cervical cancer patients in low risk group (**Figures 7A, B**). Correlation study suggested that GALNT12 negatively correlated with almost all differential immune cells, although NPL and GCNT4 did not (**Figure 7C**). NPL exhibited the strongest positive correlation with T follicular helper cell (Tfh) (r=0.51), whereas GALNT12 displayed the strongest negative association with CD56 bright natural killer cell (r=-0.5). Meanwhile, further exploration of the composition of the tumor microenvironment showed that, the Stromal, Immune, and ESTIMATE Scores were all higher in low risk group compared to high risk group, and they were all inversely connected with the risk score (**Figure 7D**). The stromal score reflected the characteristics of stromal cells in the tumor microenvironment, and higher stromal scores suggested that certain features of stromal cells were more pronounced in the low-risk group. The immune score quantified the status of immune cells in the tumor microenvironment. The higher immune score in the low-risk group indicated that the immune cell status in this group might be more favorable for combating the tumor. The ESTIMATE score, which is a composite measure of both stromal and immune cell statuses, was higher in the low-risk group, suggesting that the interaction between tumor cells and the microenvironment in this group may have been more beneficial to patient prognosis. These scores were inversely correlated with the risk score, meaning that in the low-risk group, a more favorable tumor microenvironment status was associated with a lower tumor risk. Furthermore, 34 out of 48 immunological checkpoints were substantially different between two risk groups, with all differential immune checkpoints except VTCN1 being overexpressed in low risk group (**Figure 8A**). Moreover, the TIDE analysis was used to evaluate the response of tumor patients to immunotherapy. The results showed that both Dysfunction and Exclusion scores were lower in low risk group than in high-risk group, and these two scores had different associations with risk scores, implying that cervical cancer patients in low-risk group were more likely to respond effectively to immunotherapy, though the relationship with prognostic characteristics requires further investigation (**Figures 8B, C**). Ultimately, 61 chemotherapeutic medicines (e.g., Temsirolimus and Vinorelbine) illustrated substantial differences in IC50 between two risk groups, and the top ten agents with the most notable variations might have a superior therapeutic effect on cervical cancer patients in low risk group (**Figure 8D**).

### 3.6 Prognostic genes might play a key role in cervical cancer by regulating macrophages and fibroblasts

**Figure 9:**
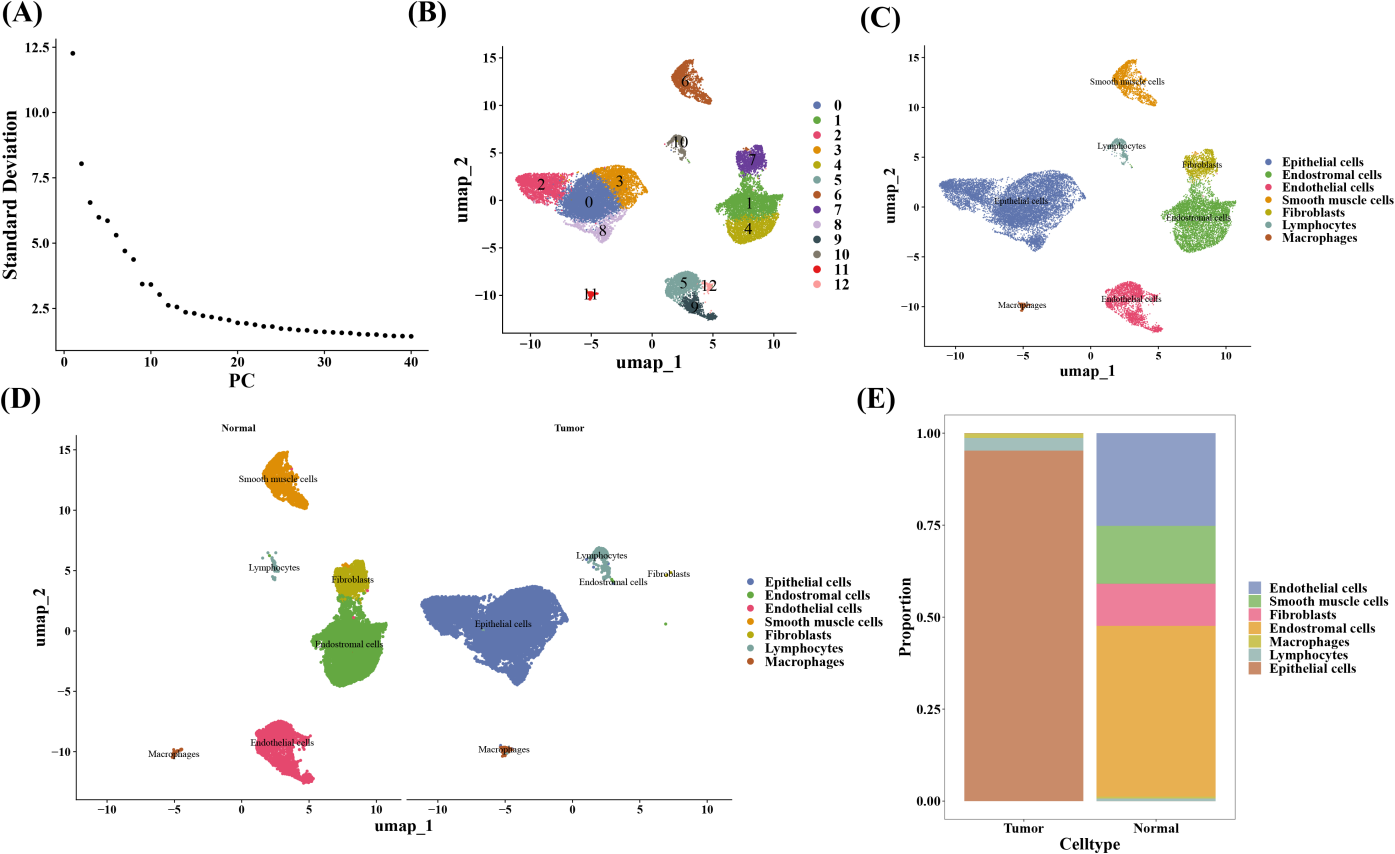
Cell annotation in single-cell analysis. **(A)** Scree plot of principal component analysis (PCA). **(B)** Clustering of UMAP cell taxa for all samples. **(C)** Cell type annotations for all samples. **(D)** Cell type annotations for both sample groups. **(E)** Proportion of each cell type.

After quality control, the scRNA-seq dataset included 20,401 cells and 22,100 genes (**Supplementary Figures 3A, B**). The top 30 PCs were chosen for UMAP clustering using high variance gene and principal component analysis (**Figures 9A**, **Supplementary Figure 3C**). The results displayed that a total of 13 clusters were included in scRNA-seq dataset (**Figures 9B**, **Supplementary Figure 3D**), and an all of 7 cell types were acquired after annotation, i.e., lymphocytes, macrophages, fibroblasts, endothelial cells, smooth muscle cells, and end ostromal (**Figures 9C, D**, **Supplementary Figure 3E**, **Supplementary Table 5**). The scale bar graph revealed that the majority cells in tumor and normal groups were epithelial cells and end ostromal cells, correspondingly (**Figure 9E**).

**Figure 10:**
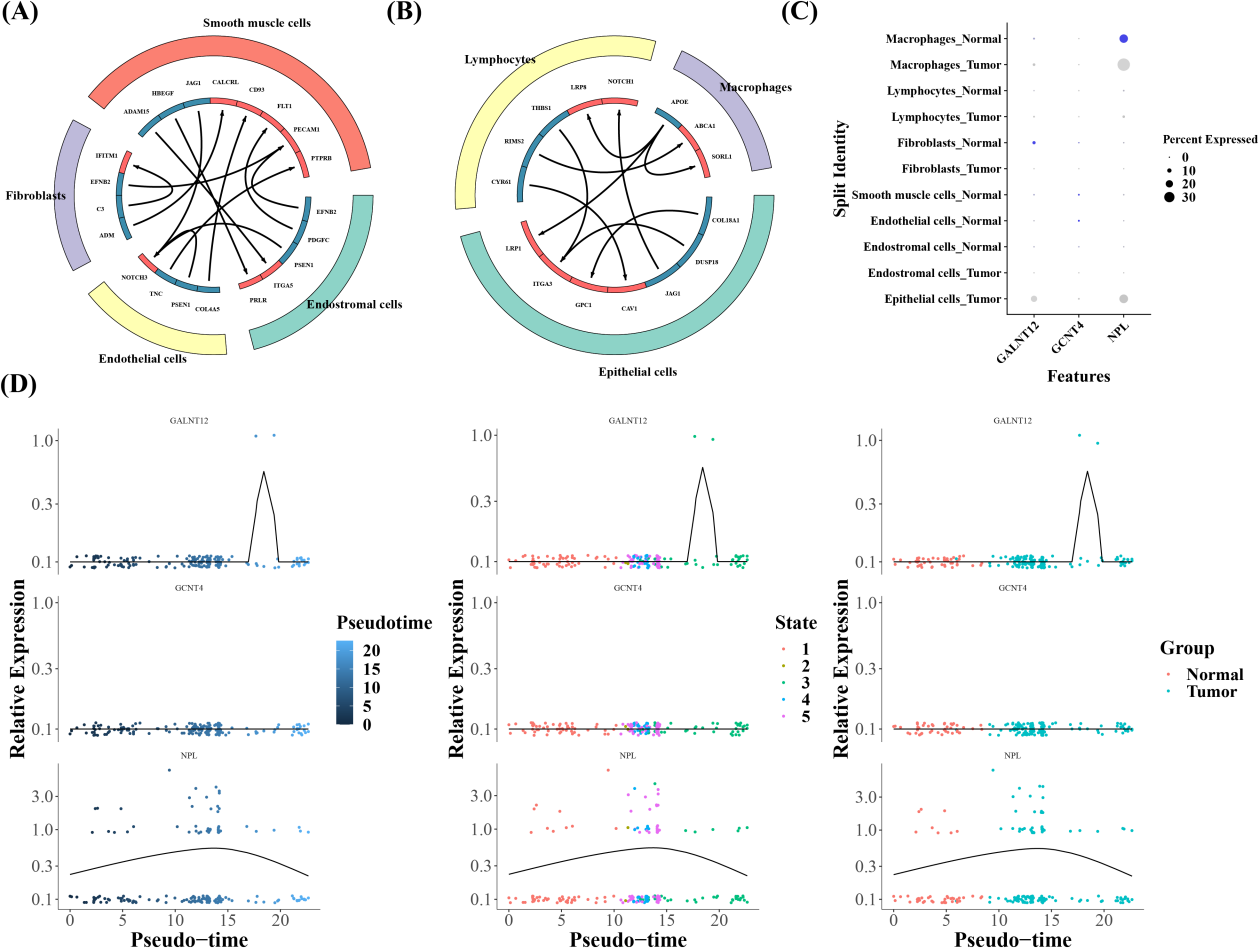
**(A, B)** Ligand-receptor interaction prediction network. The left image shows the communication interactions in Tumor cells, while the right image depicts the communication interactions in Normal cells. **(C)** Bubble map of prognostic gene expression in all cells. **(D)** Cellular temporal gene expression in Macrophages.

Furthermore, the cellular communication data suggested substantial variations in cell interactions between two groups, with lymphocytes, macrophages, and epithelial cells predominating in tumor group and the remaining four cell types populating the normal group (**Figures 10A, B**). In addition, macrophages and fibroblast cells were recognized as key cells since we saw increased expression of prognostic genes in these cells (**Figure 10C**). Nine and five differentiation states in all were discovered in fibroblasts and macrophages, according to the results of pseudotime analysis (**Supplementary Figure 4**). The analysis of temporal gene expression in cells showed that the expression of the NPL gene increased initially and then decreased during macrophage differentiation, indicating that its expression was time-dependent. This dynamic change likely reflected the activation role of NPL during the early stages of macrophage differentiation, which gradually weakened as differentiation matured. Additionally, the GALNT12 gene was highly expressed in tumor samples during the third stage of differentiation, suggesting that it might play an important role during the later stages of macrophage differentiation or in tumor-related phases (**Figure 10D**).

### 3.7 Validation of the expression of prognostic genes

**Figure 11:**
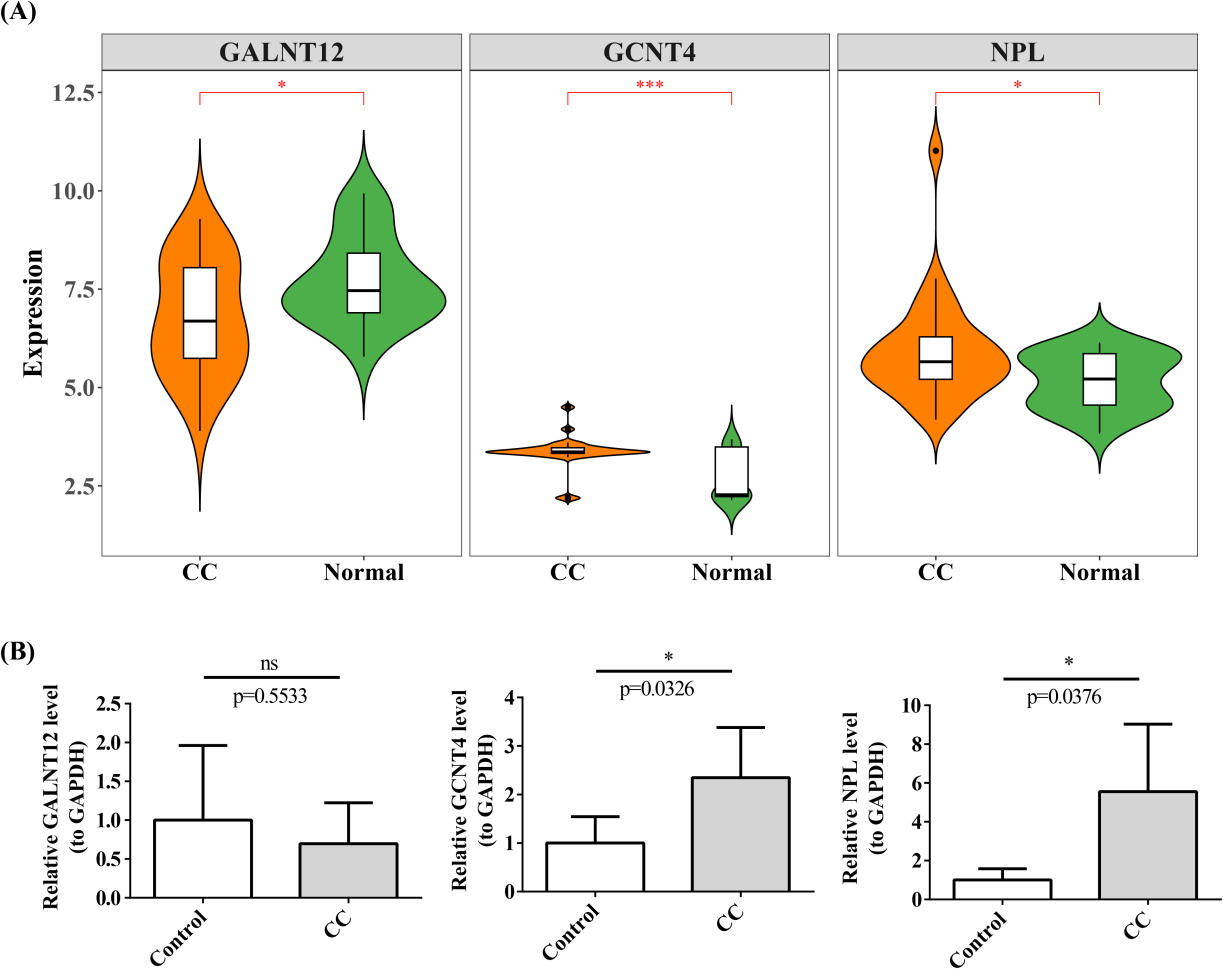
Expression of prognostic genes in single cells. **(A)** Box plot of prognostic gene expression in CC and Normal. *P < 0.05, ***P < 0.001. **(B)** Expression of prognostic genes was verified using qRT-PCR. ns represented no significance, *P < 0.05.

In GSE63514 dataset, GALNT12 expression was elevated in normal group, whereas the opposite was true for GCNT4 and NPL (**Figure 11A**). The outcomes of qRT-PCR revealed that GCNT4 and NPL were substantially increased in cervical cancer group, which corresponded to the expression trend in GSE63514 dataset (**Figure 11B**).

## 4 Discussion

Cervical cancer is one of the three most common types of gynecological malignancy, and the prognosis for advanced and recurrent cervical cancer remains poor (3). More research is needed to explore prognostic genes to guide the diagnosis, treatment, and prognosis prediction of cervical cancer. Sialylation is associated with the occurrence and development of various tumors. It affects tumor prognosis by influencing the tumor microenvironment. Research has shown that sialic acid content in cervical cancer tissues is usually higher than that in normal cervical tissues, and this abnormal sialylation is closely associated with malignancy, invasiveness, and the prognosis of cervical cancer (11). However, the relationship between sialylation and cervical cancer prognostic mechanisms has not been fully investigated. This study identified three key prognostic sialylation-related genes, GALNT12, GCNT4 and NPL, which showed significant differences in expression between cervical cancer samples and normal samples. These genes have an impact on the survival of tumor patients and on the immune infiltration and drug sensitivity of tumor cells by influencing the sialylation process of tumor cells. This provides an important reference for unravelling the pathogenesis of cervical cancer as well as improving the prognostic outcome of patients.

Three prognostic genes may influence the prognosis of cervical cancer by regulating the sialylation pathway. The GALNT12 gene encodes an enzyme called core 2 β-1,4-N-acetylgalactosaminyl transferase, which plays a crucial role in the cell and is involved in a protein modification process called O-glycosylation (37). GSEA enrichment results in this study showed that GALNT12 was significantly enriched in the O-glycan biosynthesis signaling pathway. Core 2 O-glycans are the basis for many sialylations, and the GALNT12 gene may have a significant impact on the sialylation process by catalyzing the synthesis of core 2 O-glycans, thereby affecting the prognosis of cervical cancer. Studies have shown that the expression of the GALNT12 gene is altered in several types of cancer. Additionally, GALNT12 and ST6GALNAC1 are associated within the protein-protein interaction (PPI) network, and this interaction may collaboratively regulate the sialylation process (38), further influencing tumor cell invasion, metastasis, and immune evasion (39). The results of this study are that the GALNT12 gene is a high-risk gene and has a higher expression in the high-risk group, which is consistent with previous studies. The GCNT4 gene encodes core 3 β1,3-N-acetylgalactosaminyl transferase, which plays a crucial role in cellular O-glycosylation (40). Notably, GCNT4 and GCNT3 may cooperatively regulate the structure and function of glycans through synergistic effects in the O-glycosylation process, thereby influencing tumor cell adhesion, migration, and immune evasion (41). The interaction between GCNT3 and ST3GAL1 further reveals the complex regulatory mechanisms of O-glycosylation and sialylation in tumor progression. High expression of GCNT4 has been associated with associated with poor prognosis in colorectal and breast cancer (40). But studies on gastric cancer have suggested that GCNT4 can act as a tumor suppressor and induce tumor growth arrest (40). In this study, this prognostic gene had a higher expression in the low-risk group and its high expression was associated with longer survival in patients with cervical cancer. Moreover, the GCNT4 gene was markedly enriched in the NOD-like receptor signaling pathway. As intracellular proteins, NLR proteins may be affected by O-glycosylation modifications. Abnormal expression of the GCNT4 gene may affect the function of NOD-like receptors by influencing glycosylation in the tumor microenvironment, thereby affecting tumor immune escape. The NPL gene encodes N-, a key enzyme involved in neuraminic acid metabolism (40). NPL catalyses the conversion of N-acetylneuraminic acid (sialic acid) into N-acetyl-D-mannosamine and pyruvate, thereby regulating intracellular sialic acid concentrations (40). In this study, NPL was significantly upregulated in the ubiquitin-mediated proteolysis signaling pathway, and its expression was higher in the low-risk group than in the high-risk group. One interpretation of these results could be that the high expression of the NPL gene in the low-risk group promotes the prognosis of cervical cancer patients by regulating the protein degradation signaling pathway, catalyzing the cleavage of sialic acid, and thereby reducing the expression of sialic acid in cervical cancer tissues. Furthermore, the interaction between NPL and NANP may play a crucial role in the sialylation modification of tumor cells (42), affecting tumor cell invasion, metastasis, and immune evasion (12). Future studies could further explore the specific mechanisms of NPL and NANP in cervical cancer and their potential as therapeutic targets.

A risk score model was created based on three prognostic genes to classify cervical patients into high and low risk groups. With the increase in risk score, survival time decreased, and the number of deaths increased. Through immune infiltration analysis, 19 types of immune infiltrating cells showed significant differences between the high-risk and low-risk groups, and all of them were expressed at higher levels in the low-risk group. NPL showed the strongest positive correlation with T follicular helper cells, while GALNT12 showed the highest negative correlation with correlation with CD56 bright natural killer cells. Previous studies have shown that the high expression of GALNT12 not only promotes the sialylation process but also inhibits natural killer cells, thereby suppressing anti-tumor immune responses. The high expression of the NPL gene, by cleaving sialic acid and regulating the function of NOD-like receptors, also regulates the expression of T follicular helper cells, thereby promoting anti-tumor immune responses. Therefore, these prognostic genes have the potential to serve as prognostic genes for cervical cancer. Future studies should further verify the regulatory mechanisms and clinical significance of these prognostic genes to confirm their prognostic value for cervical cancer patients. This study also found 34 immune checkpoint molecules with significant differences between the high risk group and the low risk group. The immune checkpoint VTCN1 was highly overexpressed in the high-risk group. It is speculated that tumor cells in the high-risk group upregulate the expression of this immune checkpoint molecule, thereby inhibiting the anti-tumor activity of the immune system and promoting tumor cell growth (43). This immune checkpoint may serve as a target for cervical cancer immunotherapy, and further research is needed to verify its immune escape mechanism, providing new ideas for clinical immunotherapy. A drug sensitivity analysis was performed on 138 drugs, and 10 chemotherapy drugs with the most significant differences between the high-risk and low-risk groups in IC50 were identified. The IC50 of these drugs was significantly higher in the high risk group than in the low risk group, which indicates that these chemotherapy drugs were more efficient in the high risk group. Previous studies have suggested that AP.24534 is effective in the treatment of cervical cancer patients (44). Based on the prognostic gene-based high- and low-risk grouping of cervical cancer patients, these drugs can be considered for low-risk group patients. This result has potential guiding value for the clinical use of chemotherapy drugs.

Through single-cell analysis, we found that the prognostic genes were expressed at a higher level in the macrophages and in the fibroblasts. Therefore, Macrophages and Fibroblasts were considered as the key cells in this study. As important members of the immune system, macrophages can both exert anti-tumor effects and promote tumor growth and metastasis. This dual role mainly depends on the stimulation of the tumor microenvironment and the polarization state of macrophages (45). M1 macrophages involved in anti-tumor immune responses, can directly kill tumor cells, activate T cells, and promote anti-tumor immune responses. M2 macrophages can inhibit T cell activity, promote tumor angiogenesis, and provide growth factors for tumor cells, thereby promoting tumor growth and metastasis (46, 47). In this study, the expression of the NPL gene showed a trend of first increasing and then decreasing during macrophage differentiation, while the GALNT12 gene was expressed in tumor samples during the third stage of macrophage differentiation. These findings suggest that both genes may influence tumor progression by regulating macrophage differentiation states. In the early stages of macrophage differentiation, high NPL expression may promote differentiation into an anti-tumor phenotype, thereby inhibiting tumor progression. However, as NPL expression declines, its anti-tumor function weakens, potentially facilitating tumor progression. Meanwhile, the expression of GALNT12 at specific differentiation stages may contribute to the regulation of macrophage states and indirectly affect tumor progression, though the precise regulatory mechanisms require further investigation. Fibroblasts are an important type of stromal cell in the tumor microenvironment. Through a variety of mechanisms, they can promote chemo- and radioresistance in tumor cells, such as promoting DNA repair and reducing drug concentration (48). The different expression levels of prognostic genes in different differentiation processes of key cells suggest that prognostic genes may affect the prognosis of cervical cancer by influencing the differentiation state of key cells and thus regulating anti-tumor functions.

This study utilized bioinformatics analysis based on public databases to identify key prognostic genes associated with cervical cancer, including GALNT12, GCNT4, and NPL. Preliminary findings suggest that these genes may influence tumor progression and immune evasion within the tumor microenvironment (TME) by regulating macrophage and T-cell functions. Although RT-qPCR has initially validated the expression of these key genes, extensive experimental validation is still required. Future studies should employ gene knockout techniques, cellular models, and animal models to further elucidate their precise mechanisms in cervical cancer. Additionally, assessing the clinical prognostic value of these genes through clinical trials is an important research direction. Currently, the regulatory mechanisms between prognostic genes and immune cells (such as macrophages and T cells) remain unclear. Investigating the interactions between these genes and immune cells will help uncover the complex relationship between immune cell types and the TME, offering new insights for improving cervical cancer prognosis. Notably, the area under the curve (AUC) of the constructed prognostic model in external validation was suboptimal, indicating the need for further optimization. Future studies should integrate more external datasets to refine and validate the model, enhancing its clinical applicability. In conclusion, through multi-level experimental validation and clinical translation research, this study has the potential to provide new theoretical foundations and therapeutic targets for cervical cancer prognosis assessment and treatment strategies.

This study analyzed the expression level and regulatory mechanism of sialylation in cervical cancer. Three prognostic genes related to sialylation were screened and subjected to a range of analyses, including functional enrichment, survival analysis and immune infiltrates, to demonstrate the potential clinical value of these prognostic genes in the prognosis of cervical cancer. This provides a new orientation for research on the regulatory mechanism of sialylation in cervical cancer.

## Data Availability

Publicly available datasets were analyzed in this study. This data can be found here: The datasets [ANALYZED] for this study can be found in the [GEO] [http://www.ncbi.nlm.nih.gov/geo/], [Genecards] [https://www.genecards.org/], [PubChem] [https://pubchem.ncbi.nml.gov] and [PBD] [https://www.rcsb.org/].
