# Supplementary material for: Single-cell combined transcriptome probes prognostic mechanisms of sialylation-related genes in cervical cancer
Source: Front Oncol. 2025 Apr 25;15:1534247. doi: 10.3389/fonc.2025.1534247 (PMC12061707; doi:10.3389/fonc.2025.1534247)
Supplement: Supplementary file 1 [file DataSheet1.pdf]

Supplementary Material

1 Supplementary Figures and Tables

1.1 Supplementary Figure

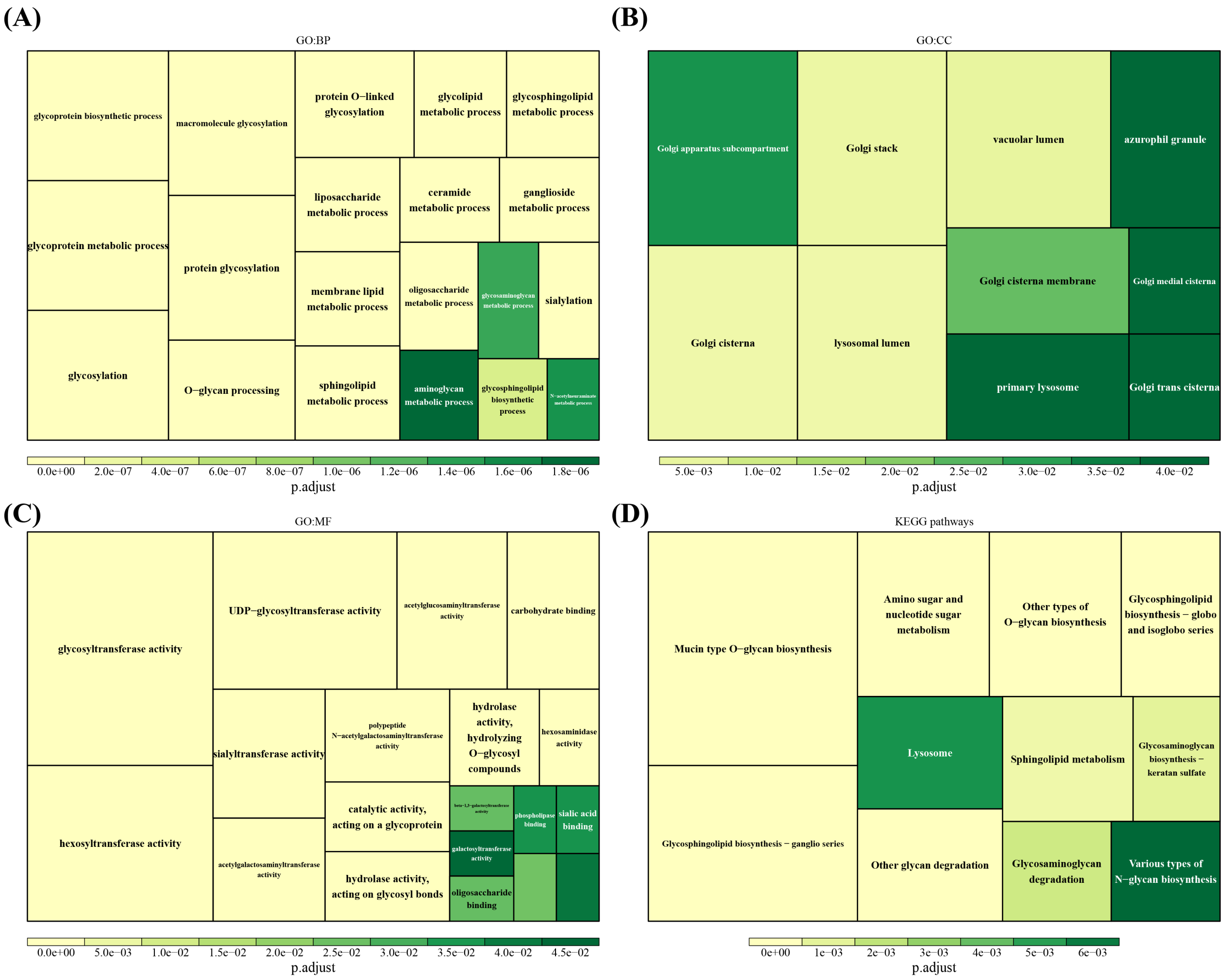

Supplementary Figure 1. Functional enrichment analysis of DE-SRGs. (A) The top 20 biological process terms enriched in DE-SRGs. **(B)** The top 20 cellular component terms enriched in DE-SRGs. **(C)** The top 20 molecular function terms enriched in DE-SRGs. **(D)** The top 20 KEGG pathways enriched in DE-SRGs.

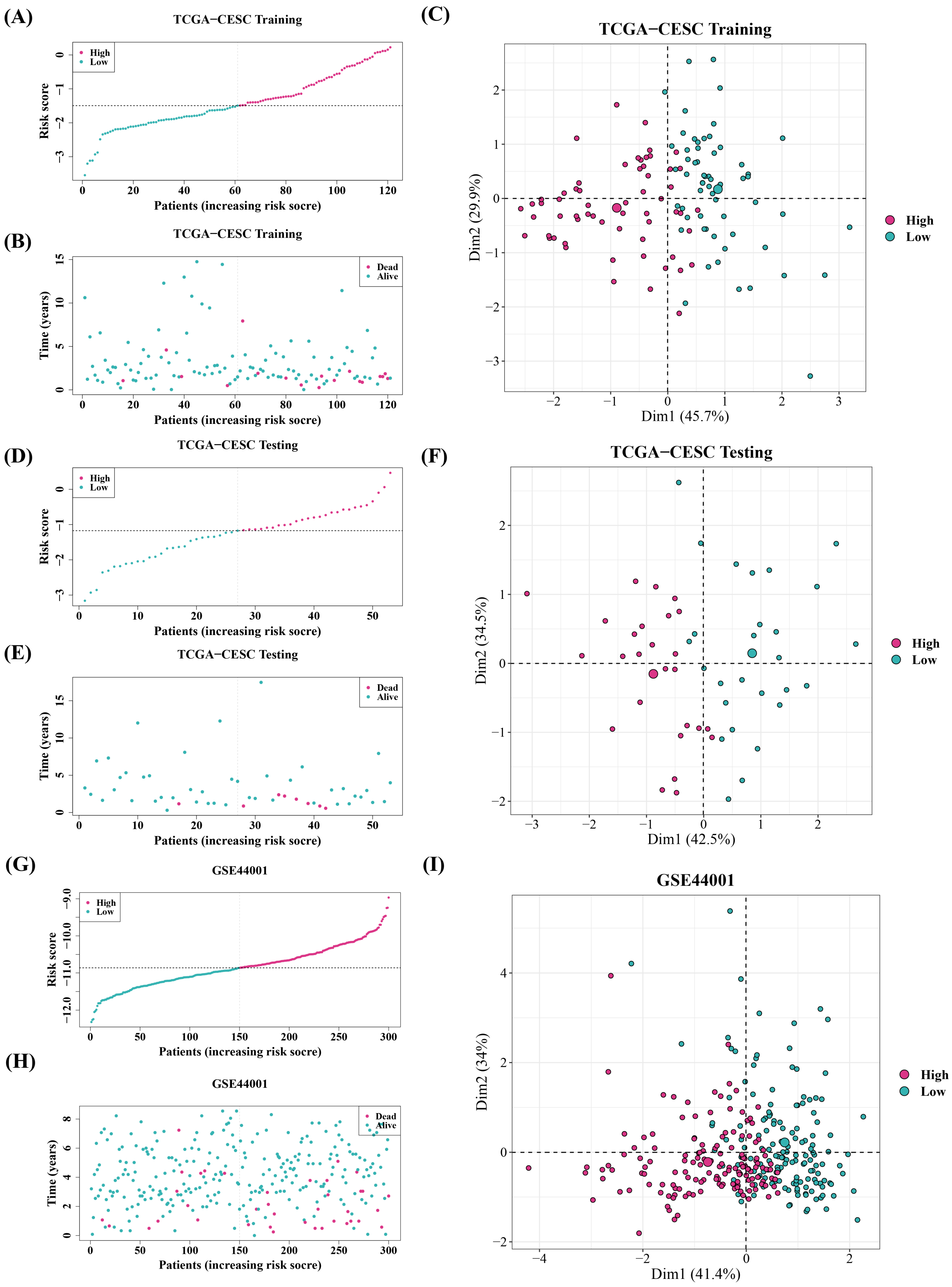
(A)
TCGA−CESC Training
High
Low
Risk score
Patients (increasing risk socre)
(B)
TCGA−CESC Training
Dead
Alive
Time (years)
Patients (increasing risk socre)
(C)
TCGA−CESC Training
Dim2 (29.9%)
Dim1 (45.7%)
High
Low
(D)
TCGA−CESC Testing
High
Low
Risk score
Patients (increasing risk socre)
(E)
TCGA−CESC Testing
Dead
Alive
Time (years)
Patients (increasing risk socre)
(F)
TCGA−CESC Testing
Dim2 (34.5%)
Dim1 (42.5%)
High
Low
(G)
GSE44001
High
Low
Risk score
Patients (increasing risk socre)
(H)
GSE44001
Dead
Alive
Time (years)
Patients (increasing risk socre)
(I)
GSE44001
Dim2 (34%)
Dim1 (41.4%)
High
Low

Supplementary Figure 2. Evaluation of risk models in training, testing, and validation sets. (A\D\G) Risk curves for high and low-risk groups. Pink points represented high-risk samples, and blue points represented low-risk samples. **(B\E\H)** Survival status of samples in high and low-risk groups. Red points represented deceased samples, and blue points represented surviving samples. **(C\F\I)** PCA plots of high and low-risk groups. Pink points represented high-risk samples, and blue points represented low-risk samples.

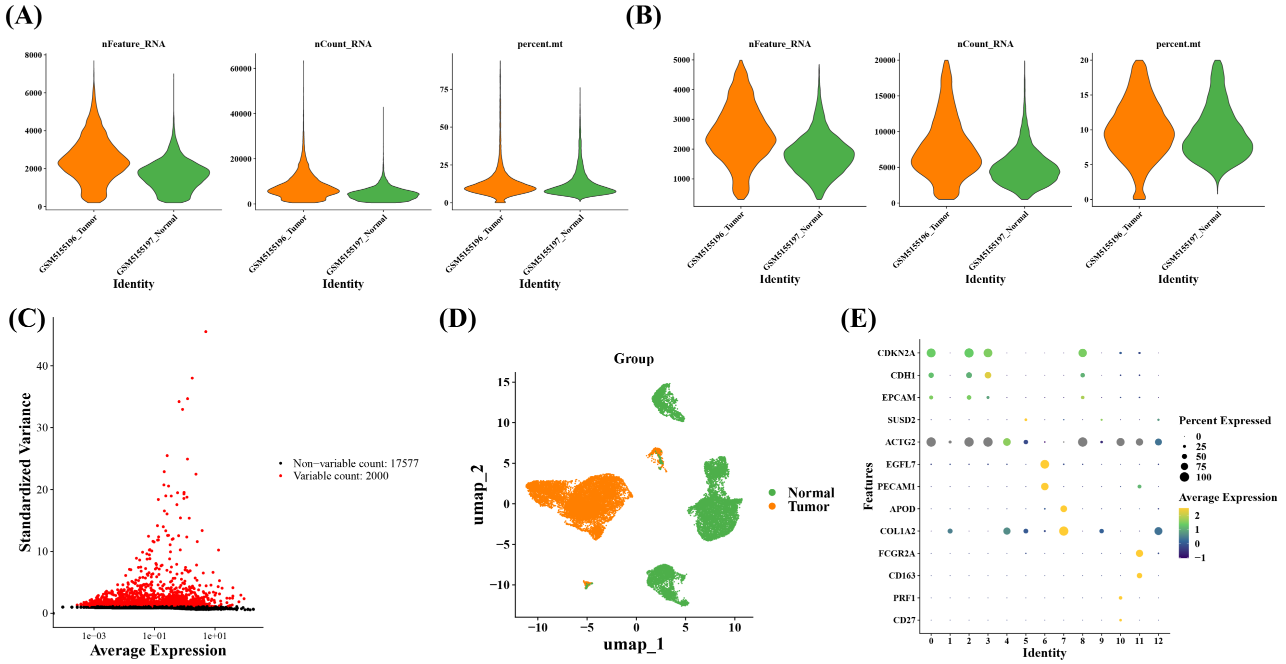

Supplementary Figure 3. Data processing of single-cell analysis. (A) Distribution plots of nFeature_RNA, nCount_RNA, and percent.mt before quality control. **(B)** Distribution plots of nFeature_RNA, nCount_RNA, and percent.mt after quality control. **(C)** Identification of the top 2000 highly variable genes. Red points represented highly variable genes, and black points represented non-highly variable genes. **(D)** UMAP clustering of cells in Tumor and Normal groups. **(E)** Expression bubble plot of marker genes across 13 different cell populations.

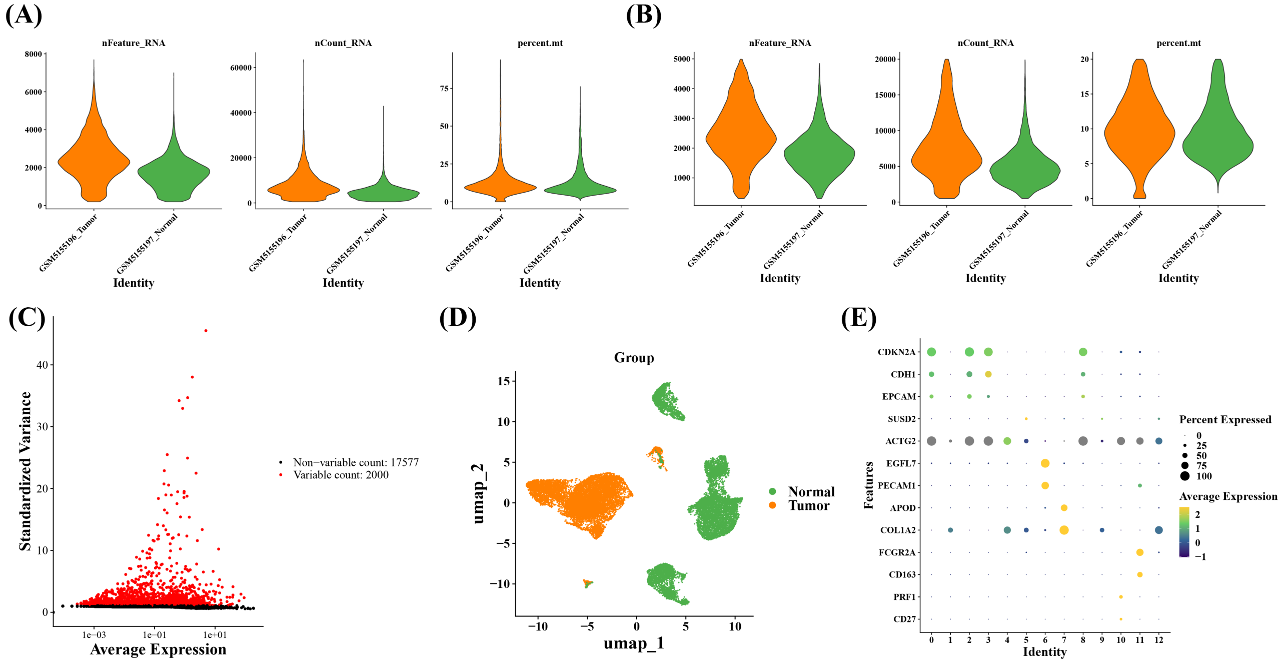

Supplementary Figure 4. The trajectory differentiation results of Macrophages and Fibroblasts cells.

1.2 Supplementary Tables.

Supplementary Table 1. List of information related to 110 sialysis related genes.

Entrez Gene ID	Gene Symbol	Pathway name
6482	ST3GAL1	1,2,3,5,6,7,8,10
6483	ST3GAL2	1,2,3,5,6,7,8,11
55808	ST6GALNAC1	1,2,5,6
51046	ST8SIA3	1,3,4,5,6,8,10
7903	ST8SIA4	1,3,4,5,6,8
8128	ST8SIA2	1,3,4,5,6,8
6487	ST3GAL3	1,3,5,6,7,8
256435	ST6GALNAC3	1,3,5,6,8

27090	ST6GALNAC4	1,3,5,6,8
30815	ST6GALNAC6	1,3,5,6,8
338596	ST8SIA6	1,3,5,6,8
81849	ST6GALNAC5	1,3,5,6,8
8869	ST3GAL5	1,3,5,6,8
10825	NEU3	1,3,9
129807	NEU4	1,3,9
4758	NEU1	1,3,9
4759	NEU2	1,3,9
6484	ST3GAL4	1,5,6,7,10
10402	ST3GAL6	1,5,6,7
10610	ST6GALNAC2	1,5,6,10
6480	ST6GAL1	1,5,6,10
29906	ST8SIA5	1,5,6
6489	ST8SIA1	1,5,6
84620	ST6GAL2	1,5,6
2720	GLB1	1,7
26503	SLC17A5	1,11,13
10020	GNE	1
10559	SLC35A1	1
140838	NANP	1

54187	NANS	1
5476	CTSA	1
55907	CMAS	1
80896	NPL	1
9334	B4GALT5	2,3,8
11226	GALNT6	2
11227	GALNT5	2
114805	GALNT13	2
117248	GALNT15	2
168391	GALNTL5	2
192134	B3GNT6	2
2589	GALNT1	2
2590	GALNT2	2
2591	GALNT3	2
26290	GALNT8	2
2650	GCNT1	2
29071	C1GALT1C1	2
374378	GALNT18	2
442117	GALNTL6	2
50614	GALNT9	2

51301	GCNT4	2
51809	GALNT7	2
55568	GALNT10	2
56913	C1GALT1	2
57452	GALNT16	2
63917	GALNT11	2
64409	GALNT17	2
79623	GALNT14	2
79695	GALNT12	2
8693	GALNT4	2
9245	GCNT3	2
140873	C20orf173	3,5,6,8
2583	B4GALNT1	3,8
8705	B3GALT4	3,8
9331	B4GALT6	3,8
2760	GM2A	3,9
3073	HEXA	3,9
3074	HEXB	3,9
20	ABCA2	3
3696	ITGB8	3
54982	CLN6	3

4099	MAG	4,12
100049587	SIGLEC14	4
114132	SIGLEC11	4
2219	FCN1	4
27036	SIGLEC7	4
27180	SIGLEC9	4
27181	SIGLEC8	4
284369	SIGLECL1	4
375790	AGRN	4
400709	SIGLEC16	4
6401	SELE	4
6403	SELP	4
8778	SIGLEC5	4
89790	SIGLEC10	4
89858	SIGLEC12	4
933	CD22	4
9370	ADIPOQ	4
945	CD33	4
946	SIGLEC6	4
10331	B3GNT3	7

10678	B3GNT2	7
11041	B4GAT1	7
11046	SLC35D2	7
23563	CHST5	7
2799	GNS	7
4166	CHST6	7
79369	B3GNT4	7
8534	CHST1	7
8702	B4GALT4	7
93010	B3GNT7	7
9435	CHST2	7
10050	SLC17A4	11,13
10246	SLC17A2	11,13
10786	SLC17A3	11,13
6568	SLC17A1	11,13
25999	CLIP3	12
54749	EPDR1	12
5660	PSAP	12
65078	RTN4R	12

1. REACTOME_SIALIC_ACID_METABOLISM

2. KEGG_O_GLYCAN_BIOSYNTHESIS

3. GOBP_GANGLIOSIDE_METABOLIC_PROCESS

4. GOMF_SIALIC_ACID_BINDING

5. GOBP_SIALYLATION

6. GOMF_SIALYLTRANSFERASE_ACTIVITY

7. GOBP_KERATAN_SULFATE_METABOLIC_PROCESS

8. GOBP_GANGLIOSIDE_BIOSYNTHETIC_PROCESS

9. GOBP_GANGLIOSIDE_CATABOLIC_PROCESS

10. GOBP_PROTEIN_SIALYLATION

11. GOBP_SIALIC_ACID_TRANSPORT

12. GOMF_GANGLIOSIDE_BINDING

13. GOMF_SIALIC_ACID_TRANSMEMBRANE_TRANSPORTER_ACTIVITY

Supplementary Table 2. A total of 4,403 differentially expressed genes (DEGs) were identified in the differential expression analysis of the GSE63514 dataset.

Supplementary Table 3. Table of GO enrichment analysis results of DE-SRGs.

ONTOLOGY	ID	Description	GeneRatio	BgRatio	pvalue	p.adjust	qvalue	geneID	Count
BP	GO:0006486	protein glycosylation	13/19	225/1889	1.72992E-21	1.77317E-19	8.74066E-20	B3GNT7/GALNT12/GALNT14/GALNT2/GALNT5/GCNT1/GCNT3/GCNT4/ST3GAL1/ST6GALNAC1/ST6GALNAC5/ST6GALNAC6/ST8SIA5	13

				03					
BP	GO:0043413	macromolecule glycosylation	13/19	225/18903	1.72992E-21	1.77317E-19	8.74066E-20	B3GNT7/GALNT12/GALNT14/GALNT2/GALNT5/GCNT1/GCNT3/GCNT4/ST3GAL1/ST6GALNAC1/ST6GALNAC5/ST6GALNAC6/ST8SIA5	13
BP	GO:0070085	glycosylation	13/19	244/18903	5.07481E-21	3.46779E-19	1.70941E-19	B3GNT7/GALNT12/GALNT14/GALNT2/GALNT5/GCNT1/GCNT3/GCNT4/ST3GAL1/ST6GALNAC1/ST6GALNAC5/ST6GALNAC6/ST8SIA5	13
BP	GO:0016266	O-glycan processing	9/19	43/18903	6.04729E-20	3.09924E-18	1.52774E-18	B3GNT7/GALNT12/GALNT14/GALNT2/GALNT5/GCNT1/GCNT3/GCNT4/ST3GAL1	9
BP	GO:0009101	glycoprotein biosynthetic process	13/19	321/18903	1.89785E-19	7.7812E-18	3.83566E-18	B3GNT7/GALNT12/GALNT14/GALNT2/GALNT5/GCNT1/GCNT3/GCNT4/ST3GAL1/ST6GALNAC1/ST6GALNAC5/ST6GALNAC6/ST8SIA5	13
BP	GO:0009100	glycoprotein metabolic process	13/19	395/18903	2.88449E-18	9.85533E-17	4.85808E-17	B3GNT7/GALNT12/GALNT14/GALNT2/GALNT5/GCNT1/GCNT3/GCNT4/ST3GAL1/ST6GALNAC1/ST6GALNAC5/ST6GALNAC6/ST8SIA5	13

BP	GO:0006493	protein O-linked glycosylation	9/19	90/18903	7.40547E-17	2.16874E-15	1.06906E-15	B3GNT7/GALNT12/GALNT14/GALNT2/GALNT5/GCNT1/GCNT3/GCNT4/ST3GAL1	9
BP	GO:0001573	ganglioside metabolic process	6/19	27/18903	1.25285E-13	3.20281E-12	1.57879E-12	HEXA/HEXB/NEU1/ST3GAL1/ST6GALNAC5/ST6GALNAC6	6
BP	GO:0006687	glycosphingolipid metabolic process	7/19	62/18903	1.40611E-13	3.20281E-12	1.57879E-12	HEXA/HEXB/NEU1/ST3GAL1/ST6GALNAC5/ST6GALNAC6/ST8SIA5	7
BP	GO:0006664	glycolipid metabolic process	7/19	105/18903	6.35347E-12	1.26707E-10	6.24589E-11	HEXA/HEXB/NEU1/ST3GAL1/ST6GALNAC5/ST6GALNAC6/ST8SIA5	7
BP	GO:1903509	liposaccharide metabolic process	7/19	106/18903	6.79891E-12	1.26707E-10	6.24589E-11	HEXA/HEXB/NEU1/ST3GAL1/ST6GALNAC5/ST6GALNAC6/ST8SIA5	7

BP	GO:0097503	sialylation	5/19	21/18903	1.1655E-11	1.99107E-10	9.81476E-11	ST3GAL1/ST6GALNAC1/ST6GALNAC5/ST6GALNAC6/ST8SIA5	5
BP	GO:0009311	oligosaccharide metabolic process	6/19	64/18903	3.10467E-11	4.89583E-10	2.41335E-10	HEXB/NEU1/ST6GALNAC1/ST6GALNAC5/ST6GALNAC6/ST8SIA5	6
BP	GO:0006665	sphingolipid metabolic process	7/19	163/18903	1.43856E-10	2.10646E-09	1.03836E-09	HEXA/HEXB/NEU1/ST3GAL1/ST6GALNAC5/ST6GALNAC6/ST8SIA5	7
BP	GO:0006672	ceramide metabolic process	6/19	107/18903	7.29584E-10	9.97098E-09	4.91509E-09	HEXA/HEXB/NEU1/ST3GAL1/ST6GALNAC5/ST6GALNAC6	6
BP	GO:0006643	membrane lipid metabolic process	7/19	209/18903	8.2238E-10	1.05367E-08	5.19398E-09	HEXA/HEXB/NEU1/ST3GAL1/ST6GALNAC5/ST6GALNAC6/ST8SIA5	7

BP	GO:0006688	glycosphingolipid biosynthetic process	4/19	34/18903	3.31609E-08	3.99881E-07	1.97117E-07	ST3GAL1/ST6GALNAC5/ST6GALNAC6/ST8SIA5	4
BP	GO:0030203	glycosaminoglycan metabolic process	5/19	123/18903	1.16188E-07	1.32325E-06	6.52284E-07	B3GNT7/GALNT5/HEXA/HEXB/ST3GAL1	5
BP	GO:0006054	N-acetylneuraminate metabolic process	3/19	11/18903	1.41328E-07	1.52486E-06	7.51663E-07	NANP/NPL/ST3GAL1	3
BP	GO:0006022	aminoglycan metabolic process	5/19	134/18903	1.78307E-07	1.82764E-06	9.00919E-07	B3GNT7/GALNT5/HEXA/HEXB/ST3GAL1	5
BP	GO:0046479	glycosphingolipid catabolic process	3/19	15/18903	3.88735E-07	3.79479E-06	1.8706E-06	HEXA/HEXB/NEU1	3

BP	GO:0001574	ganglioside biosynthetic process	3/19	17/18903	5.80228E-07	5.1716E-06	2.54929E-06	ST3GAL1/ST6GALNAC5/ST6GALNAC6	3
BP	GO:0019377	glycolipid catabolic process	3/19	17/18903	5.80228E-07	5.1716E-06	2.54929E-06	HEXA/HEXB/NEU1	3
BP	GO:0009247	glycolipid biosynthetic process	4/19	72/18903	7.18072E-07	6.13354E-06	3.02346E-06	ST3GAL1/ST6GALNAC5/ST6GALNAC6/ST8SIA5	4
BP	GO:0006024	glycosaminoglycan biosynthetic process	4/19	77/18903	9.41557E-07	7.72077E-06	3.80587E-06	B3GNT7/GALNT5/HEXA/ST3GAL1	4
BP	GO:0006023	aminoglycan biosynthetic process	4/19	81/18903	1.15463E-06	9.10378E-06	4.48761E-06	B3GNT7/GALNT5/HEXA/ST3GAL1	4
BP	GO:0046	ceramide catabolic process	3/19	22/189	1.30988E	9.94539E	4.90248E	HEXA/HEXB/NEU1	3

	514			03	-06	-06	-06		
BP	GO:1903510	mucopolysaccharide metabolic process	4/19	95/18903	2.18977E-06	1.60323E-05	7.90294E-06	B3GNT7/HEXA/HEXB/ST3GAL1	4
BP	GO:0009312	oligosaccharide biosynthetic process	3/19	28/18903	2.77587E-06	1.96226E-05	9.67274E-06	ST6GALNAC1/ST6GALNAC5/ST6GALNAC6	3
BP	GO:0030148	sphingolipid biosynthetic process	4/19	111/18903	4.07776E-06	2.77221E-05	1.36653E-05	ST3GAL1/ST6GALNAC5/ST6GALNAC6/ST8SIA5	4
BP	GO:0030149	sphingolipid catabolic process	3/19	32/18903	4.19213E-06	2.77221E-05	1.36653E-05	HEXA/HEXB/NEU1	3
BP	GO:0046466	membrane lipid catabolic process	3/19	37/18903	6.54628E-06	4.19371E-05	2.06725E-05	HEXA/HEXB/NEU1	3

BP	GO:0006040	amino sugar metabolic process	3/19	41/18903	8.95834E-06	5.56503E-05	2.74322E-05	NANP/NPL/ST3GAL1	3
BP	GO:0046467	membrane lipid biosynthetic process	4/19	149/18903	1.3106E-05	7.90213E-05	3.89528E-05	ST3GAL1/ST6GALNAC5/ST6GALNAC6/ST8SIA5	4
BP	GO:1901136	carbohydrate derivative catabolic process	4/19	176/18903	2.52365E-05	0.000147814	7.28633E-05	HEXA/HEXB/NEU1/NPL	4
BP	GO:0046513	ceramide biosynthetic process	3/19	68/18903	4.13999E-05	0.000235749	0.000116221	ST3GAL1/ST6GALNAC5/ST6GALNAC6	3
BP	GO:0016051	carbohydrate biosynthetic process	4/19	212/18903	5.22281E-05	0.000289372	0.000142643	B3GNT7/ST6GALNAC1/ST6GALNAC5/ST6GALNAC6	4

BP	GO:1903238	positive regulation of leukocyte tethering or rolling	2/19	12/18903	6.27953E-05	0.000338764	0.000166699	GCNT1/SELE	2
BP	GO:0009313	oligosaccharide catabolic process	2/19	14/18903	8.64776E-05	0.000454562	0.000224072	HEXB/NEU1	2
BP	GO:0018146	keratan sulfate biosynthetic process	2/19	16/18903	0.000113 9	0.000569499	0.000280729	B3GNT7/ST3GAL1	2
BP	GO:0030214	hyaluronan catabolic process	2/19	16/18903	0.000113 9	0.000569499	0.000280729	HEXA/HEXB	2
BP	GO:0060993	kidney morphogenesis	3/19	97/18903	0.000119575	0.000583639	0.000287699	GCNT1/GCNT3/GCNT4	3
BP	GO:1903 03	regulation of leukocyte tethering or rolling	2/19	17/189	0.000129	0.000615	0.000303	GCNT1/SELE	2

	236			03	009	043	179		
BP	GO:0042339	keratan sulfate metabolic process	2/19	18/18903	0.000145048	0.000675793	0.000333125	B3GNT7/ST3GAL1	2
BP	GO:1904996	positive regulation of leukocyte adhesion to vascular endothelial cell	2/19	25/18903	0.000283217	0.001290213	0.000635997	GCNT1/SELE	2
BP	GO:0006027	glycosaminoglycan catabolic process	2/19	30/18903	0.000409437	0.001824663	0.000899449	HEXA/HEXB	2
BP	GO:0050901	leukocyte tethering or rolling	2/19	33/18903	0.000496079	0.002163748	0.001066597	GCNT1/SELE	2
BP	GO:0030212	hyaluronan metabolic process	2/19	35/18903	0.000558359	0.002384657	0.001175492	HEXA/HEXB	2

BP	GO:0006026	aminoglycan catabolic process	2/19	36/18903	0.000590849	0.002471921	0.001218508	HEXA/HEXB	2
BP	GO:1904994	regulation of leukocyte adhesion to vascular endothelial cell	2/19	40/18903	0.000729777	0.002992086	0.001474918	GCNT1/SELE	2
BP	GO:0002691	regulation of cellular extravasation	2/19	41/18903	0.000766742	0.003082003	0.001519241	GCNT1/SELE	2
BP	GO:0050885	neuromuscular process controlling balance	2/19	50/18903	0.001139281	0.004491395	0.002213987	HEXA/HEXB	2
BP	GO:0044242	cellular lipid catabolic process	3/19	222/18903	0.00134762	0.005212491	0.002569443	HEXA/HEXB/NEU1	3
BP	GO:0061	leukocyte adhesion to	2/19	57/18	0.001478	0.005611	0.002766	GCNT1/SELE	2

	756	vascular endothelial cell		903	112	353	058		
BP	GO:0045123	cellular extravasation	2/19	74/18903	0.002476173	0.009229371	0.004549523	GCNT1/SELE	2
BP	GO:0019915	lipid storage	2/19	87/18903	0.003403041	0.012414764	0.00611973	HEXA/HEXB	2
BP	GO:0001822	kidney development	3/19	309/18903	0.00345191	0.012414764	0.00611973	GCNT1/GCNT3/GCNT4	3
BP	GO:0072001	renal system development	3/19	318/18903	0.003742004	0.01322605	0.006519645	GCNT1/GCNT3/GCNT4	3
BP	GO:0007040	lysosome organization	2/19	98/18903	0.004295278	0.014673156	0.007232981	HEXA/HEXB	2

BP	GO:0080171	lytic vacuole organization	2/19	98/18903	0.004295278	0.014673156	0.007232981	HEXA/HEXB	2
BP	GO:0016042	lipid catabolic process	3/19	336/18903	0.004366159	0.014673156	0.007232981	HEXA/HEXB/NEU1	3
BP	GO:0001655	urogenital system development	3/19	360/18903	0.005292168	0.017498298	0.008625605	GCNT1/GCNT3/GCNT4	3
BP	GO:0042552	myelination	2/19	142/18903	0.008812288	0.028549727	0.014073293	HEXA/HEXB	2
BP	GO:0007272	ensheathment of neurons	2/19	144/18903	0.009052353	0.028549727	0.014073293	HEXA/HEXB	2

BP	GO:0008366	axon ensheathment	2/19	144/18903	0.009052353	0.028549727	0.014073293	HEXA/HEXB	2
BP	GO:0044272	sulfur compound biosynthetic process	2/19	148/18903	0.009541256	0.029635718	0.014608621	B3GNT7/ST3GAL1	2
BP	GO:0018242	protein O-linked glycosylation via serine	1/19	10/18903	0.010008345	0.030622549	0.015095069	GALNT2	1
BP	GO:0050905	neuromuscular process	2/19	154/18903	0.010296373	0.030972072	0.015267363	HEXA/HEXB	2
BP	GO:0016052	carbohydrate catabolic process	2/19	155/18903	0.010424746	0.030972072	0.015267363	HEXB/NEU1	2

BP	GO:0007605	sensory perception of sound	2/19	159/18903	0.010945391	0.03177195	0.015661654	HEXA/HEXB	2
BP	GO:0046349	amino sugar biosynthetic process	1/19	11/18903	0.011003944	0.03177195	0.015661654	NANP	1
BP	GO:0050954	sensory perception of mechanical stimulus	2/19	180/18903	0.013863285	0.038742371	0.019097651	HEXA/HEXB	2
BP	GO:0046348	amino sugar catabolic process	1/19	14/18903	0.013985051	0.038742371	0.019097651	NPL	1
BP	GO:0090715	immunological memory formation process	1/19	14/18903	0.013985051	0.038742371	0.019097651	ST3GAL1	1

BP	GO:0007626	locomotory behavior	2/19	197/18903	0.016446188	0.044952914	0.022159075	HEXA/HEXB	2
BP	GO:0090713	immunological memory process	1/19	17/18903	0.016957644	0.045741013	0.02254756	ST3GAL1	1
BP	GO:0006044	N-acetylglucosamine metabolic process	1/19	18/18903	0.017946462	0.046570343	0.022956369	NANP	1
BP	GO:0006677	glycosylceramide metabolic process	1/19	18/18903	0.017946462	0.046570343	0.022956369	ST6GALNAC6	1
BP	GO:0050884	neuromuscular process controlling posture	1/19	18/18903	0.017946462	0.046570343	0.022956369	HEXA	1
BP	GO:0002	leukocyte migration involved in	1/19	19/189	0.018934	0.048215	0.023767	SELE	1

	523	inflammatory response		03	654	979	569		
BP	GO:0007033	vacuole organization	2/19	213/18903	0.019051192	0.048215979	0.023767569	HEXA/HEXB	2
BP	GO:0001780	neutrophil homeostasis	1/19	20/18903	0.019921745	0.049204311	0.024254757	GCNT4	1
BP	GO:0006491	N-glycan processing	1/19	20/18903	0.019921745	0.049204311	0.024254757	ST8SIA5	1
CC	GO:0043202	lysosomal lumen	3/19	98/19869	0.000106464	0.003297758	0.002041956	HEXA/HEXB/NEU1	3
CC	GO:0031985	Golgi cisterna	3/19	120/19869	0.000193986	0.003297758	0.002041956	GALNT2/GCNT1/ST3GAL1	3

CC	GO:0005795	Golgi stack	3/19	153/19869	0.00039629	0.004491284	0.002780981	GALNT2/GCNT1/ST3GAL1	3
CC	GO:0005775	vacuolar lumen	3/19	176/19869	0.000596439	0.005069733	0.003139153	HEXA/HEXB/NEU1	3
CC	GO:0032580	Golgi cisterna membrane	2/19	92/19869	0.003445287	0.023427951	0.014506471	GALNT2/ST3GAL1	2
CC	GO:0098791	Golgi apparatus subcompartment	3/19	387/19869	0.005634816	0.031930625	0.019771285	GALNT2/GCNT1/ST3GAL1	3
CC	GO:0005766	primary lysosome	2/19	155/19869	0.009477671	0.038821695	0.024038201	HEXA/HEXB	2

CC	GO:0042582	azurophil granule	2/19	155/19869	0.009477671	0.038821695	0.024038201	HEXA/HEXB	2
CC	GO:0000138	Golgi trans cisterna	1/19	12/19869	0.011418146	0.038821695	0.024038201	ST3GAL1	1
CC	GO:0005797	Golgi medial cisterna	1/19	12/19869	0.011418146	0.038821695	0.024038201	ST3GAL1	1
MF	GO:0016757	glycosyltransferase activity	15/19	267/18432	6.43493E-25	1.67308E-23	4.74153E-24	B3GNT7/GALNT12/GALNT14/GALNT2/GALNT5/GCNT1/GCNT3/GCNT4/HEXA/HEXB/ST3GAL1/ST6GALNAC1/ST6GALNAC5/ST6GALNAC6/ST8SIA5	15
MF	GO:0008194	UDP-glycosyltransferase activity	10/19	142/18432	4.64986E-17	6.04482E-16	1.71311E-16	B3GNT7/GALNT12/GALNT14/GALNT2/GALNT5/GCNT1/GCNT3/GCNT4/HEXA/HEXB	10

MF	GO:0016758	hexosyltransferase activity	10/19	195/18432	1.18445E-15	1.02652E-14	2.90918E-15	B3GNT7/GALNT12/GALNT14/GALNT2/GALNT5/GCNT1/GCNT3/GCNT4/HEXA/HEXB	10
MF	GO:0008375	acetylglucosaminyltransferase activity	6/19	43/18432	2.97224E-12	1.93196E-11	5.47518E-12	B3GNT7/GCNT1/GCNT3/GCNT4/HEXA/HEXB	6
MF	GO:0008373	sialyltransferase activity	5/19	21/18432	1.32191E-11	6.87392E-11	1.94807E-11	ST3GAL1/ST6GALNAC1/ST6GALNAC5/ST6GALNAC6/ST8SIA5	5
MF	GO:0004653	polypeptide N-acetylgalactosaminyltransferase activity	4/19	19/18432	3.09447E-09	1.34094E-08	3.80023E-09	GALNT12/GALNT14/GALNT2/GALNT5	4
MF	GO:0008376	acetylgalactosaminyltransferase activity	4/19	34/18432	3.66649E-08	1.36184E-07	3.85946E-08	GALNT12/GALNT14/GALNT2/GALNT5	4
MF	GO:0140	catalytic activity, acting on a glycoprotein	3/19	23/184	1.62326E	5.27561E	1.49511E	GCNT1/GCNT3/GCNT4	3

	103			32	-06	-06	-06		
MF	GO:0030246	carbohydrate binding	5/19	275/18432	6.9842E-06	2.01766E-05	5.71806E-06	GALNT12/GALNT14/GALNT2/GALNT5/SELE	5
MF	GO:0015929	hexosaminidase activity	2/19	15/18432	0.000104863	0.000272644	7.72674E-05	HEXA/HEXB	2
MF	GO:0004553	hydrolase activity, hydrolyzing O-glycosyl compounds	3/19	94/18432	0.000117231	0.000277277	7.85807E-05	HEXA/HEXB/NEU1	3
MF	GO:0016798	hydrolase activity, acting on glycosyl bonds	3/19	132/18432	0.000319871	0.000693054	0.000196412	HEXA/HEXB/NEU1	3
MF	GO:0008499	UDP-galactose:beta-N-acetylglucosamine beta-1,3-galactosyltransferase activity	1/19	12/18432	0.012303553	0.024607105	0.006973674	B3GNT7	1

MF	GO:0048531	beta-1,3-galactosyltransferase activity	1/19	15/1843	0.015356956	0.026618724	0.007543768	B3GNT7	1
MF	GO:0070492	oligosaccharide binding	1/19	15/1843	0.015356956	0.026618724	0.007543768	SELE	1
MF	GO:0033691	sialic acid binding	1/19	22/1843	0.022446825	0.035873434	0.011016656	SELE	1
MF	GO:0043274	phospholipase binding	1/19	23/1843	0.023455707	0.035873434	0.011016656	SELE	1
MF	GO:0035250	UDP-galactosyltransferase activity	1/19	28/1843	0.028485336	0.041145486	0.011660664	B3GNT7	1
MF	GO:000808	galactosyltransferase activity	1/19	31/184	0.031491	0.04309339	0.012212	B3GNT7	1

378	32	323	702

Supplementary Table 4. Table of KEGG enrichment analysis results of DE-SRGs.

ID	Description	GeneRatio	BgRatio	pvalue	p.adjust	qvalue	geneID	Count
hsa00512	Mucin type O-glycan biosynthesis	9/19	36/8644	1.14355E-17	2.17274E-16	9.62988E-17	GALNT12/GALNT14/GALNT2/GALNT5/GCNT1/GCNT3/GCNT4/ST3GAL1/ST6GALNAC1	9
hsa00604	Glycosphingolipid biosynthesis - ganglio series	6/19	15/8644	2.3208E-13	2.20476E-12	9.77177E-13	HEXA/HEXB/ST3GAL1/ST6GALNAC5/ST6GALNAC6/ST8SIA5	6
hsa00514	Other types of O-glycan biosynthesis	4/19	47/8644	2.80138E-06	1.57626E-05	6.98619E-06	GALNT12/GALNT14/GALNT2/GALNT5	4
hsa00520	Amino sugar and nucleotide sugar metabolism	4/19	49/8644	3.31844E-06	1.57626E-05	6.98619E-06	HEXA/HEXB/NANP/NPL	4
hsa00603	Glycosphingolipid biosynthesis - globo and isoglobo series	3/19	16/8644	4.95249E-06	1.88195E-05	8.34104E-06	HEXA/HEXB/ST3GAL1	3
hsa00511	Other glycan degradation	3/19	18/8644	7.19646E-06	2.27888E-05	1.01003E-05	HEXA/HEXB/NEU1	3

hsa00600	Sphingolipid metabolism	3/19	54/8644	0.000208077	0.00056478	0.000250318	HEXA/HEXB/NEU1	3
hsa00533	Glycosaminoglycan biosynthesis - keratan sulfate	2/19	14/8644	0.000410064	0.000973903	0.000431647	B3GNT7/ST3GAL1	2
hsa00531	Glycosaminoglycan degradation	2/19	19/8644	0.000765527	0.001616112	0.000716282	HEXA/HEXB	2
hsa04142	Lysosome	3/19	132/8644	0.002820168	0.00535832	0.002374879	HEXA/HEXB/NEU1	3
hsa00513	Various types of N-glycan biosynthesis	2/19	42/8644	0.003740143	0.006460247	0.002863267	HEXA/HEXB	2

Supplementary Table 5. Seven cell types were identified after annotation.

Celltype	Tumor	Normal	All
Endostromal cells	6	4464	4470
Endothelial cells	0	2421	2421
Epithelial cells	10280	0	10280
Fibroblasts	2	1098	1100
Lymphocytes	373	70	443
Macrophages	130	46	176

Smooth muscle cells	0	1511	1511
